# Variations of the Virome in Raw and Treated Water: A One‐Year Follow‐Up at Six Different Drinking Water Treatment Plants

**DOI:** 10.1111/1758-2229.70222

**Published:** 2025-10-28

**Authors:** Fredy Saguti, Hao Wang, Marianela Patzi Churqui, Timur Tunovic, Linda Holmer, Ämma Pettersson, Caroline Schleich, Britt‐Marie Pott, Olof Bergstedt, Kristina Nyström, Heléne Norder

**Affiliations:** ^1^ Department of Infectious Diseases Institute of Biomedicine, University of Gothenburg Gothenburg Sweden; ^2^ Görvälnverket, Norrvatten Järfälla Sweden; ^3^ Nodra Norrköping Sweden; ^4^ Vatten & Miljö i Väst AB Falkenberg Sweden; ^5^ Applied Microbiology, Department of Chemistry Lund University Lund Sweden; ^6^ Sydvatten AB Malmö Sweden; ^7^ Göteborgs Stad Kretslopp Och Vatten Gothenburg Sweden

**Keywords:** drinking water treatment plant, metagenomics, virome

## Abstract

Little is known about virome changes in raw and drinking water over time, and differences between raw water sources and treatment technologies. This study used metagenomics to assess viruses prevalent in raw and drinking water samples over 1 year from six Swedish drinking water treatment plants (DWTPs) with varying treatment barriers and with different raw water sources. Sequences homologous to known viruses in the raw water samples were detected by amplification and next‐generation sequencing and classified into 152 different virus species belonging to 76 virus families/orders. The majority were small bacteriophages. Other viral genomes were homologous to viruses infecting plants, invertebrates, vertebrates, mammals and giant viruses infecting amoeba or algae. Several virus species were simultaneously found in both raw and drinking water, indicating passage through the purification barriers, although reduced by 1–3 log_10_ after treatment. Most viruses detected in water samples after ultrafiltration were small viruses, and other barriers appeared more effective at removing smaller viruses. To avoid detecting viruses possibly replicating within DWTPs, viruses were separated according to the possibility that the host could be found in the water sources or not. These results underscore the importance of monitoring both raw and drinking water for small viruses, especially when viral contamination of the source water is at risk, to ensure drinking water quality.

## Graphical abstract

This paper describes monitoring of outgoing water from drinking water treatment plants, geographically spread over half of Sweden and sampled every other month for 1 year. We found differences in the virome from the different water sources from each plant as well as differences in virus size and seasonal variation, where we also could detect viruses in the drinking water despite different treatment strategies in the different plants. This demonstrates the importance of analyzing viruses in the water from treatment plants, especially if they change treatment techniques.

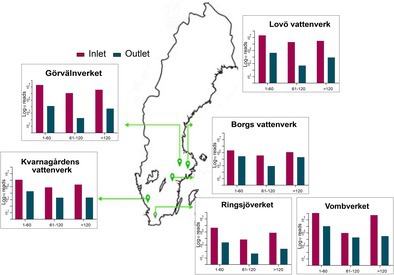

## 1 Introduction

Water safety depends on sufficient reduction of chemical and biological contaminants, such as infectious pathogens including viruses, during the purification of raw water in drinking water treatment plants (DWTPs). The drinking water treatment systems in Swedish DWTPs use several barriers to treat raw water into drinking water. Most plants are equipped with conventional barriers, such as flocculation, sedimentation, different types of filtrations (rapid and slow sand filtrations, and carbon filtration), and chlorine disinfection. Many also use high‐tech treatment barriers such as ozonation, UV light, nanofiltration and ultrafiltration as additional barriers. DWTPs rely on groundwater, artificial groundwater and surface water from lakes and rivers as a source of their raw water. Treatment of raw water presents many new challenges for DWTPs as the quality of surface water changes in connection with climate change and extreme hydrological events (Delpla et al. 2009). The most vulnerable surface waters are related to shallow lakes where changes in water temperature can alter their pH and chemical composition (Delpla et al. 2009; Moreira and Bondelind 2017). Additionally, heavy rains can cause flooding that risks contaminating surface water with waterborne pathogens from livestock or other animals living near the water, and/or from broken or overflowing sewer lines (Chalmers 2012).

Viruses are intracellular parasites and need their cellular host for replication. They are ubiquitous and found in all forms of water used for drinking water production such as surface water (lakes and rivers) and groundwater (Yates et al. 1985; Pinon and Vialette 2018). Groundwater is the most biologically stable water; it contains few viruses and is least affected by environmental changes, as shown by measuring chemical and physical parameters over time, especially if there is no intrusion from surface water (Espinosa et al. 2008). The stability of viruses, and thereby potentially their infectivity, in water differs (Pinon and Vialette 2018). Those belonging to the *Caliciviridae*, *Adenoviridae* and *Picornaviridae* virus families have been reported to infect humans after 42 to 61 days in the aquatic environment (Seitz et al. 2011; Kotwal and Cannon 2014). Traditionally, viruses have been identified in different waters by virus isolation in cell cultures (Böttiger and Herrström 1992; Figas et al. 2017).

For the removal of viruses from water, there is a demand for accurate data on the barriers that DWTPs use for the disinfection of the water (Carol et al. 2021; Kauppinen et al. 2019; Chen et al. 2016). Normally, bench or pilot‐scale studies are conducted to assess the performance of various barriers used at DWTPs to remove viruses from water. These small‐scale pilot studies investigate the virus efficiency of water treatment barriers by spiking known virus concentrations in water. The virus concentrations are determined by isolation in cell cultures or quantification by qPCR (Saguti et al. 2022, 2023). Small‐scale pilot studies allow for detailed monitoring of the treatment barriers, but do not control for all the factors involved in the full‐scale DWTP. Some studies have investigated viral removal performances under full‐scale operation conditions in DWTPs with only one raw water source (Wang et al. 2020; Asami et al. 2016), or studying different raw water conditions or hydrometeorological events, such as heavy rainfall, flooding or snow melting (Asami et al. 2016; Sylvestre et al. 2021).

The water quality indicators used today to identify possible viruses or other microorganisms are mainly molecular PCR‐based analyses, with quantitative PCR being the most common method due to its high sensitivity (Saguti et al. 2021; Albinana‐Gimenez et al. 2009). However, these PCR‐based analyses are limited by the requirement of prior knowledge of the genomes of the pathogens being investigated (Shen et al. 2024). Without prior knowledge of which viruses are present in what levels in the water in the DWTPs, quantitative PCR analysis is not feasible. Also, monitoring many microorganisms simultaneously is difficult with quantitative PCR, as one set of primers and probes is needed for each microorganism. Today, the use of high‐throughput metagenomics sequencing has led to the discovery of novel human‐associated viruses including non‐culturable pathogenic viruses in wastewater (Bibby et al. 2019).

Little is known about the variation of the virome and the temporal and qualitative variations of treatment performance over a longer period of time at DWTPs using different barriers and raw water sources. In this study, we investigated the virome change in raw and drinking water every 2 months for a year at six Swedish DWTPs with different raw water sources and differences in the treatment processes. Metagenomic sequencing was used to detect and identify the virome in the raw water and the drinking water, providing useful information about which viruses are detected in high concentrations in different sources of water. A further goal of this study was to estimate how the virome of different raw water sources varied over time, and if any variation in the amount of viruses affected the effectiveness of different treatment processes in removing viruses.

## 2 Materials and Methods

### 2.1 Drinking Water Treatment Companies and Sampling Sites

**FIGURE 1:**
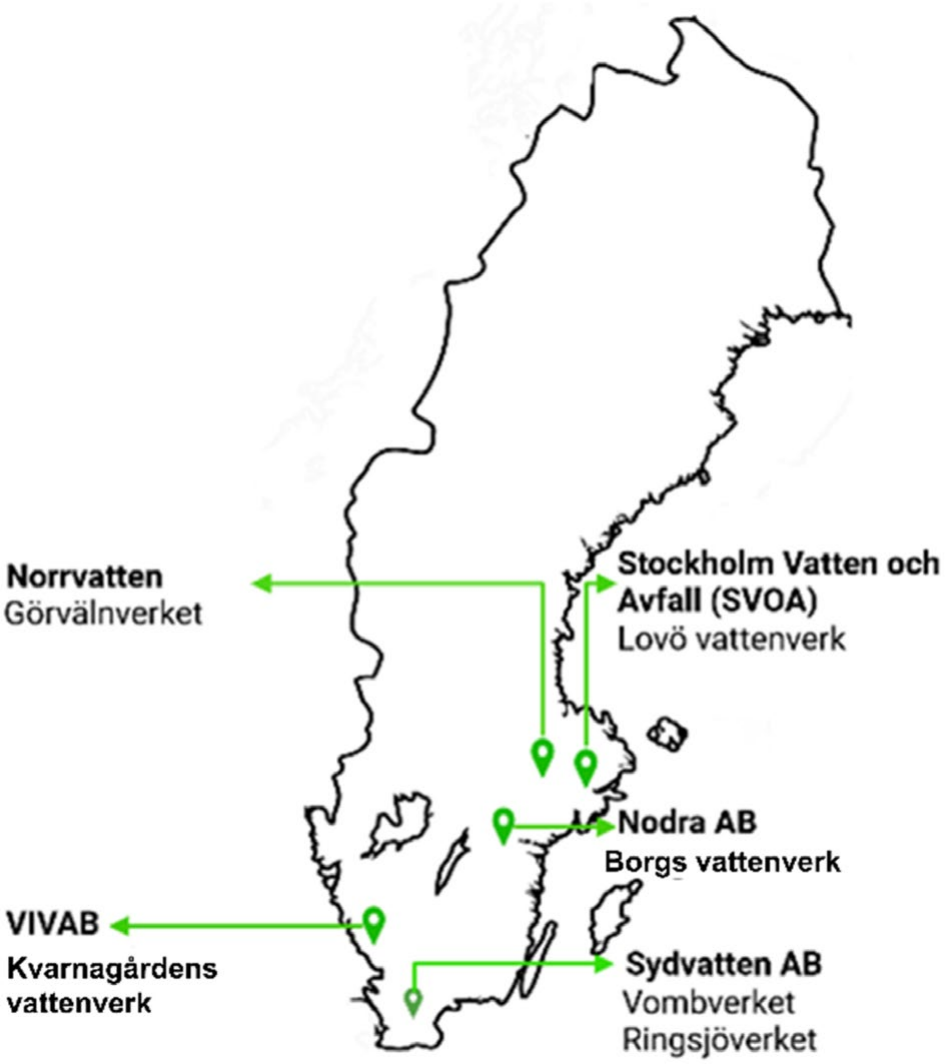
A map of the locations of the five drinking water companies with their six DWTPs where water samples were collected in the southern part of Sweden.

Five drinking water companies in the southern part of Sweden participated in this study. They were Stockholm Vatten och Avfall (SVOA), Norrvatten, Nodra AB, Vatten och Miljö i Väst AB (Vivab) and Sydvatten AB. Water samples were collected from six DWTPs: Lovö vattenverk owned by SVOA, Görvälnverket under Norrvatten, Borgs vattenverk under Nodra AB, Kvarnagårdens vattenverk owned by Vivab and Vombverket and Ringsjöverket, both owned by Sydvatten AB (Figure 1).

**FIGURE 2:**
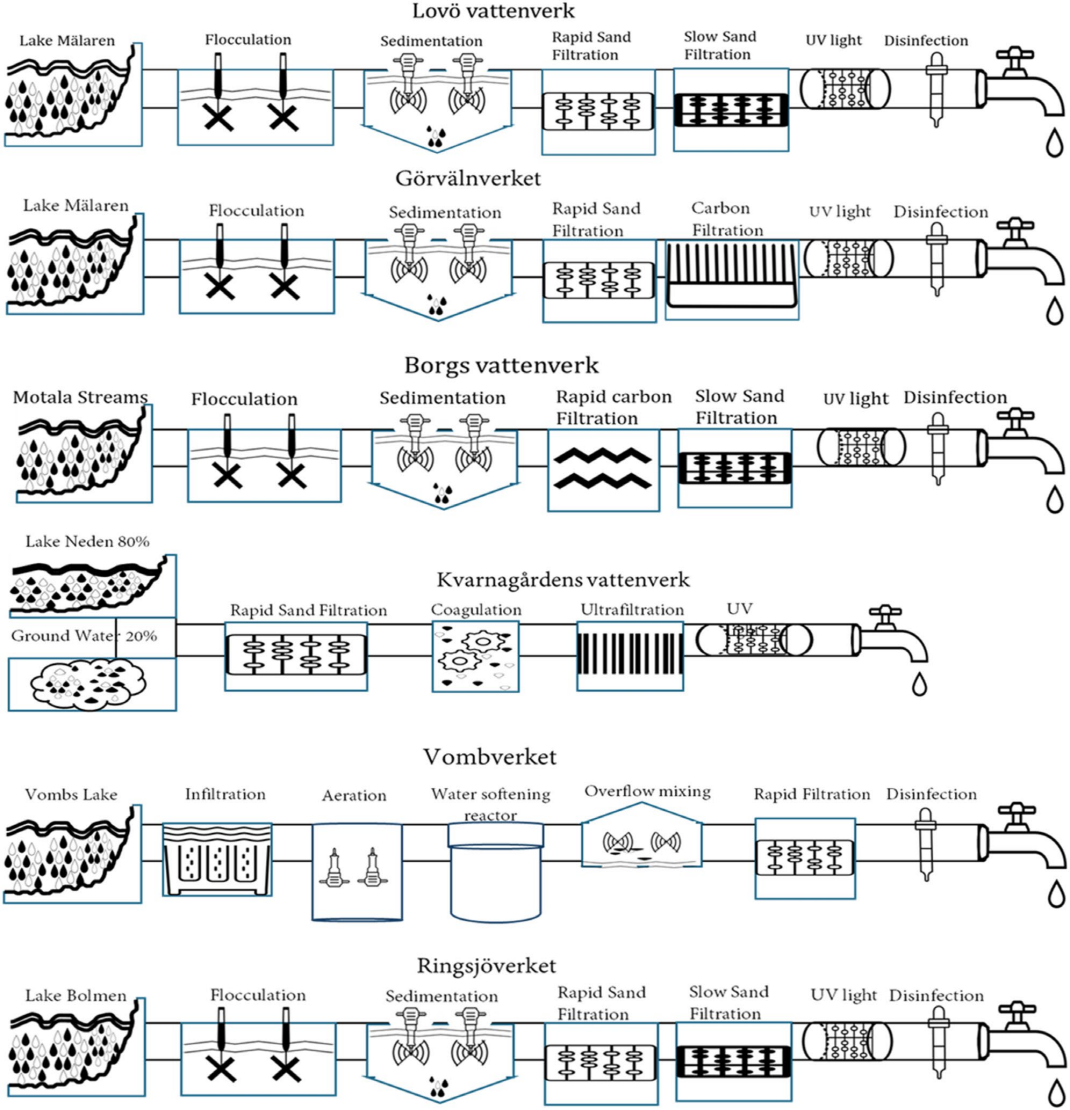
A schematic representation of the different purification processes used at the six DWTPs (Lovö vattenverk, Görvälnverket, Borgs vattenverk, Kvarnagårdens vattenverk, Vombverket and Ringsjöverket) included in the study.

The DWTPs are installed with multiple barriers which are mainly filters such as infiltration, flocculation, rapid sand filters, slow sand filters and ultrafiltration (Figure 2, Table S1). At Lovö vattenverk and Görvälnverkets DWTPs, microbial disinfection of the water is mainly performed by UV light followed by low doses of monochloramine to reduce the re‐growth of microorganisms during distribution. Both UV light and monochloramine are used as the main disinfection methods against microorganisms at Borgs vattenverk. At Kvarnagårdens vattenverk, the removal of microorganisms from the raw water is primarily performed by ultrafiltration combined with coagulation after rapid sand filtration followed by viral inactivation by UV light. Vombverket uses infiltration after raw water passes through sieve filters. The viral disinfectant is monochloramine. At Ringsjöverket, both UV light and hypochlorite are used to disinfect the water from viruses and other microorganisms before it is distributed for consumption.

Raw and drinking water samples were collected between March 2021 and April 2022, with the intention of collecting samples every 2 months, that is, six of each water sample were planned to be collected from each DWTP throughout the study period (Table S2). At all DWTPs, purified drinking water was collected by connecting Nano‐Ceram cartridge filters (Argonide, Sanford, Florida, USA) to the effluent lines, and the filtered water volumes were recorded (Table S2). The Nano‐Ceram cartridge filters, with a pore size of 2 μm, utilize a highly electropositive filter membrane media to absorb small particles. All but two DWTPs, Görvälnverket and Lovö vattenverk, also connected a Nano‐Ceram filter to the incoming raw water pipes. They calculated the flow rate of the water in the DWTP to allow the same water that had been sampled at the inlet to be sampled after treatment. They determined the flow rate and volume of purified water passing through the filter. Immediately after the collection, the Nano‐Ceram filters, through which raw and purified water had passed, were shipped to the Clinical Microbiological Laboratory (CML) at Sahlgrenska University Hospital in Gothenburg. Görvälnverket and Lovö vattenverk sent 20 L of raw water to CML instead of Nano‐Ceram filters, due to clogging of the filters, not permitting collection with Nano‐Ceram filters on site. Filters were shipped on ice and 20 L samples were shipped at room temperature, both arriving within 24 h. The virus concentration from all water samples was performed as previously described (Wang et al. 2020). Briefly, viruses were eluted from the Nano‐Ceram filter with 0.2 M phosphate buffer containing 0.05 M glycine and 3% beef extract (pH 9.5), the eluate was filtered through a 0.65/0.45 μm Sartobran Capsule (Sartorius, Göttingen, Germany) with a double layer cellulose membrane, one of each pore size, to remove bacteria. The filtrate was then ultracentrifuged at 50,000 rpm for 4 h, and the pellet was resuspended in 2.4 mL Tris–HCl per sample. All results were calculated in relation to the volume of water collected by the DWTPs.

### 2.2 Extraction of Nucleic Acid Material and Amplification by PCR

RNA was extracted from 200 μL of concentrated sample by using the PureLink RNA Mini kit (ThermoFisher Scientific, Carlsbad, CA, USA) according to the manufacturer's instructions. After extraction, the viral RNA was eluted with 100 μL RNase‐free water. RNA extracts were immediately used as a template to prepare cDNA using the High Capacity cDNA Reverse Transcription kit (ThermoFisher Scientific) according to the manufacturer's instructions. The PureLink Genomic DNA mini kit (ThermoFisher Scientific) was used for the extraction of DNA from the 200 μL of the same samples according to the manufacturer's instructions. After extraction, the viral DNA was eluted with 100 μL PureLink genomic elution buffer. The PCR and preparation of library templates for NGS were performed as previously described (Wang et al. 2020), where random primers were used for PCR amplification of both cDNA and DNA separately. The products were sequenced on the Illumina NovSeq X plus platform (Novogene, Cambridge, UK) with paired‐end sequencing, generating reads of 150 bp.

### 2.3 NGS Data Analysis

Raw data from the Illumina sequencing was imported to CLC Genomic Workbench 23.0.5 (Qiagen, Hilden, Germany) for analysis. The sequenced reads under a quality score of 0.05 were processed. The primer and adapter sequences were removed by trimming, and all reads of 150 bp or longer were accepted. The reads were de novo assembled into contigs using a CLC de novo assembler with a word size of 20 and an automatic bubble size of 50 bp. After that, the contigs with consensus lengths above 500 bp were blasted against a locally built‐in genomic viral database in the CLC Genomic Workbench using all viral sequences from the National Center for Biotechnology Information (NCBI) GenBank with BLASTn (Altschul et al. 1990). For accepted possible viral hits, the cut‐off for the *E* value was < 10^−5^, and High Scoring Segment Pairs (HSP) length > 500 bp was used to further analyze all possible viral sequences.

In parallel, assembled contig FASTA files were analysed with additional platforms. On the **Galaxy** web platform (https://usegalaxy.eu), **Kraken2**, a taxonomic sequence classifier that assigns sequences by comparison to reference databases, was employed against the 2019 virus genome database along with a prebuilt virus reference index (version: 2022‐06‐07, downloaded 2022‐08‐04). Contigs were also analysed using the **NMDC EDGE** web‐based platform (https://nmdc‐edge.org) through the “single metagenomic” and “Viruses & Plasmids” workflows (Kelliher et al. 2024). These pipelines integrate **geNomad** for microbial and viral sequence detection and include **CheckV** to evaluate genome quality, completeness and contamination. Only viral assignments with a nucleotide identity ≥ 70% were retained.

Using only one of the techniques could lead to false positive hits. Therefore, for downstream analysis, we used longer contigs in BLASTn and Kraken2, and only accepted those genomes with at least 100 nucleotide homology to reference viral sequences. Analyses were limited to known viral sequences in GenBank, rather than all sequences found in the NCBI. For validation, two viral genomes (Syngen and BeAn viruses) were used to map raw reads from all water samples, and in each case, contigs identified by BLAST and Kraken2 had reads mapping across the full viral genomes. All sequenced samples produced viral hits confirmed by both BLAST and Kraken2 (Table S3).

The identified viral contigs were further classified into virus family level, size and host using ICTV data for subsequent analysis. The classified viruses were divided into three groups based on their size, 1–60 nm, 61–120 nm and > 121 nm, as most human viruses range from 18 to 200 nm in size, using data from ICTV (Lefkowitz et al. 2018) (Table S4). If the size of a virus was unknown, a size range of the virus family was used. The smallest diameter of viruses that are rod‐shaped or have differences in length and width was used as its size. This was because viruses are flexible, and protein molecules can get through narrow pores even if part of the virion is larger than the pore itself. The rationale for size categorization is based on the fact that viruses of different sizes behave differently in the water treatment process. Smaller viruses (1–60 nm) are often difficult to remove because they can pass through many filter membranes (Wang et al. 2020). The virus size range of 61–120 nm was assumed to effectively be removed by DWTPs with ultrafiltration membrane pore sizes 20–40 nm. The larger viruses > 121 nm were assumed to be removed effectively by most treatment barriers. The viral contigs were further divided into three groups based on the type of host of the virus and its association with the aquatic environment (Table S4). Group 1: viruses infecting hosts that may be present in the treatment process; Group 2: viruses infecting hosts unlikely to be present in the treatment process; Group 3: viruses infecting hosts not present in the treatment process. The reason for this grouping was that viruses with possible hosts present during the treatment of the water may replicate and thereby multiply; the determination of the purification from these viruses may therefore be uncertain.

### 2.4 NGS Sequencing Calculations

The estimated number of viral genomes per 1000 L of water Wn, was calculated to normalise viral abundance based on the volume of water collected and to allow comparison across samples. The formula used was, $\mathrm{Wn}=\left(\left(N\times F\right)/V\right)\times 1000$, n is the type of water sample collected (raw water or drinking water), N is the number of identified viral genomes confirmed by BLASTn, and Kraken2 or geNomad in the water sample. F is the dilution factor of the virus genomes in the water sample analysed and V is the volume of the water sample collected for analysis from the collection site. Dilution factor F is calculated by $F=J\left[\frac{m}{\left(l\times s\right)}\right]$, J is the 2.4 mL of diluted pellets after ultracentrifugation, m is 200 μL of diluted pellets used for extraction of nucleic acid materials, l is 30 μL used for preparation of Illumina library and s is 1/50, the dilution factor for the volume used in PCR amplification from nested PCRs.

The estimated number of viral genomes of the different viruses in the water samples analysed was used to calculate the log reduction of viral contigs from raw water to drinking water. The log_10_ reduction was calculated by ${\mathrm{Log}}_{10}\;\text{Reduction}={\mathrm{Log}}_{10}\mathrm{No}-{\mathrm{Log}}_{10}\mathrm{Nt}$, whereby No is the estimated number of viral contigs in raw water, and Nt is the estimated number of viral contigs in the drinking water samples.

Significant differences in the reduction of contigs between inlet and outlet samples were assessed using the Mann–Whitney test. *p* values were corrected for multiple testing using the Benjamini‐Hochberg false discovery rate (FDR), with tests having an FDR < 0.05 considered significant. Additionally, *t*‐tests assuming unequal variance were assessed on certain parametric data. All statistical analyses were performed using GraphPad Prism 10 (GraphPad Software Inc., San Diego, CA, USA).

## 3 Results

### 3.1 NGS Analysis for Detection of Viruses in the Water Samples

**FIGURE 3:**
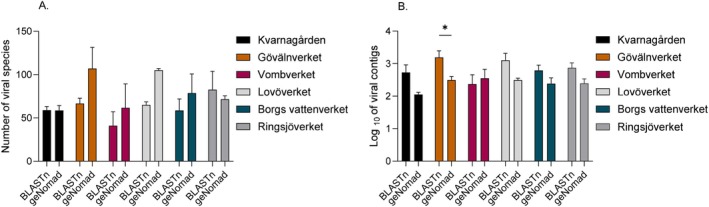
Number of viral species identified using BLASTn in CLC and geNomad across drinking water treatment plants, presented for inlet (raw water). The panel shows (A) number of viral species and (B) the log10 viral contigs identified per sample.

Across all water treatment plants, both the BLASTn and geNomad detected relatively similar numbers of viral species, with geNomad identifying slightly more species, but the difference was not significant and no greater than two fold (Figure 3A). In contrast, Figure 3B shows that BLASTn identifies higher numbers of viral contigs, particularly in the Görvalnverket treatment plant. This may reflect BLASTn's approach in classifying more fragmented and partial viral sequences, leading to higher total contig counts but not necessarily to higher species richness. We continued the analysis with BLASTn viral contigs.

The NGS analysis of the samples from Vombverket and Ringsjöverket showed a low quality of the extracted nucleic acids in the samples from May/June and also from September/October in samples from Ringsjöverket; therefore, only five pairs of samples from Vombverket and four from Ringsjöverket were included in the analysis from these DWTPs (Table S3). The average number of total reads obtained varied from 1.09 × 10^9^ per 1000 L of drinking water collected from Görvälnverket to 8.28 × 10^12^ per 1000 L of raw water collected from Lovö vattenverk (Table S3).

The mean number of contigs longer than 500 nucleotides varied from 2.3 × 10^5^ to 4.9 × 10^9^ in the raw water samples and 4.85 × 10^4^ to 3.4 × 10^7^ in the drinking water samples per 1000 L (Table S3). These contigs were analysed for viral sequences, and less than 2% of the contigs were confirmed as viral sequences and varied from 1.7 × 10^5^ to 1.5 × 10^7^ per 1000 L in the raw water and 2.9 × 10^3^ to 4.7 × 10^4^ per 1000 L in the drinking water samples (Table S3).

The DWTPs using raw water from Lake Mälaren (Lovö vattenverk and Görvälnverket) and Lake Vomb (Vombverket) had a higher number of viral contigs in the raw water samples compared to the other DWTPs (Tables 1 and 2). The reduction of mean number of viral contigs from raw water to drinking water was most efficient in Lovö vattenverk and Görvälnverkets DWTPs, while Borgs DWTP had the least number of viral contigs identified in the raw water and the least reduction of the contigs of all DWTPs (Tables 1 and 2). This DWTP relies on Lake Glan as a source of raw water and collects its raw water at Motala River 3 km from Lake Glan.

**TABLE 1:** The mean number of viral contigs based on the size by NGS analysis for the raw water (Inlet/1000 L) and drinking water (Outlet/1000 L) of the six DWTPs and their log_10_ reduction.

DWTPs	Size of viruses (nm)	Inlet/1000 L	Outlet/1000 L	Log_10_ Reduction	p‐value
Lovö vattenverk	1–60	8,786,967	24,749	2.55	0.01*
	61–120	912,144	350	3.42	0.89
	> 121	933,400	5658	2.22	0.07^a^
	Total	10,632,511	30,758	2.54	
Görvälnverket	1–60	11,953,453	9499	3.10	0.02*
	61–120	753,533	159	3.68	0.11
	> 121	2,366,873	3975	2.77	0.05^a^
	Total	15,073,860	13,632	3.04	
Borgs vattenverk	1–60	101,008	14,315	0.85	0.59
	61–120	18,679	560	1.52	0.01*
	> 121	55,898	10,497	0.73	0.55
	Total	175,584	25,372	0.84	
Kvarnagårdens vattenverk	1–60	552,853	12,100	1.66	0.002** ^,^ ^a^
	61–120	43,764	1424	1.49	0.02* ^,^ ^a^
	> 121	111,827	1491	1.87	0.71
	Total	708,443	15,016	1.67	
Vombverket	1–60	1,512,098	41,818	1.56	0.31
	61–120	6703	1976	0.53	0.31
	> 121	856,432	2876	2.47	0.007**
	Total	2,375,233	46,670	1.71	
Ringsjöverket	1–60	461,326	2571	2.25	0.01* ^,^ ^a^
	61–120	7176	51	2.15	0.01* ^,^ ^a^
	> 121	78,596	270	2.46	0.03* ^,^ ^a^
	Total	547,098	2891	2.28	

* *p* < 0.05.

** *p* < 0.01.

^a^ Usage of a parametric test. *

**TABLE 2:** The mean number of viral contigs per 1000 L water. The contigs were classified into three groups based on hosts.

DWTPs	Virus hosts	Inlet	Outlet	Log_10_ Reduction	*p*
Lovö vattenverk	1 Phages replicate during purification process	7,687,040	25,242	2.48	0.06^a^
	2 Phages with hosts not found in purification process	900,060	3900	2.36	0.08^a^
	3 Viruses without host in purification process	1,793,487	2350	2.88	0.93
	Total	10,380,587	31,492	2.52	
Görvälnverket	1 Phages replicate during purification process	11,280,040	12,796	2.95	0.06
	2 Phages with hosts not found in purification process	1,700,120	967	3.24	0.04* ^,^ ^a^
	3 Viruses without host in purification process	2,260,200	454	3.70	0.29
	Total	15,240,360	14,217	3.03	
Borgs vattenverk	1 Phages replicate during purification process	180,500	24,449	0.87	0.24
	2 Phages with hosts not found in purification process	18,685	4429	0.63	0.81
	3 Viruses without host in purification process	16,815	802	1.32	0.93
	Total	215,999	29,680	0.86	
Kvarnagårdens vattenverk	1 Phages replicate during purification process	564,388	11,384	1.70	0.06
	2 Phages with hosts not found in purification process	59,201	2246	1.42	0.01*
	3 Viruses without with host in purification	64,392	1223	1.72	0.04*
	Total	687,982	14,854	1.67	
Vombverket	1 Phages replicate during purification process	1,218,675	37,426	1.51	0.01*
	2 Phages with hosts not found in purification process	306,171	11,245	1.44	0.22
	3 Viruses without host in purification process	1,000,455	3264	2.49	0.03*
	Total	2,525,301	51,936	1.69	
Ringsjöverket	1 Phages replicate during purification process	246,247	1654	2.17	0.2
	2 Phages with hosts not found in purification process	61,092	698	1.94	0.03*
	3 Viruses without host in purification process	89,940	270	2.52	0.03*
	Total	397,279	2621	2.18	

*Note:* Contigs with sequences homologous to viruses with possible hosts present during the purification process were classified into Group 1. Contigs with sequences homologous to bacteriophages with hosts probably not present during the purification process were classified into Group 2. Contigs with sequences homologous to viruses without hosts present in the purification process were classified into Group 3.

* *p* < 0.05.

^a^ Usage of a parametric test.

Even though the number of viral contigs was lower in the drinking water samples than in raw water, the number of different virus species identified did not differ substantially between the water samples (Table S3, Figure S1). There were contigs homologous to sequences of 1 to 76 different viruses in the raw water samples and 4 to 47 different viruses in the drinking water samples. The contigs were in total homologous to 152 different virus species classified into 76 virus families or groups. Both the size and the host were known for 152 of the classified virus species (Table S4). The majority of these viruses (74/152) were bacteriophages; the others were viruses infecting plants (4/152), arthropods (14/152), invertebrates (12/152), vertebrates (11/152), mammals (27/152), giant viruses infecting amoeba or algae (9/152) and their virophages (1/152). The host, but not the size, was known for the remaining five classified viruses.

### 3.2 Water Treatment Purification Efficiency in Relation to Virus Size

The confirmed virus contigs representing viruses classified into the 76 different virus families were divided into three groups based on virus size, 1–60 nm, 61–120 nm and > 120 nm (Table S4). There were differences in the relative amount of viruses of different sizes, with the smallest viruses (< 61 nm) being the most common. The average numbers of contigs homologous to these viruses per 1000 L of the analysed samples varied between 1.0 × 10^5^ and 1.2 × 10^7^ in raw water, and 2.6 × 10^3^ and 4.2 × 10^4^ in the treated drinking water during the year (Table 1).

**FIGURE 4:**
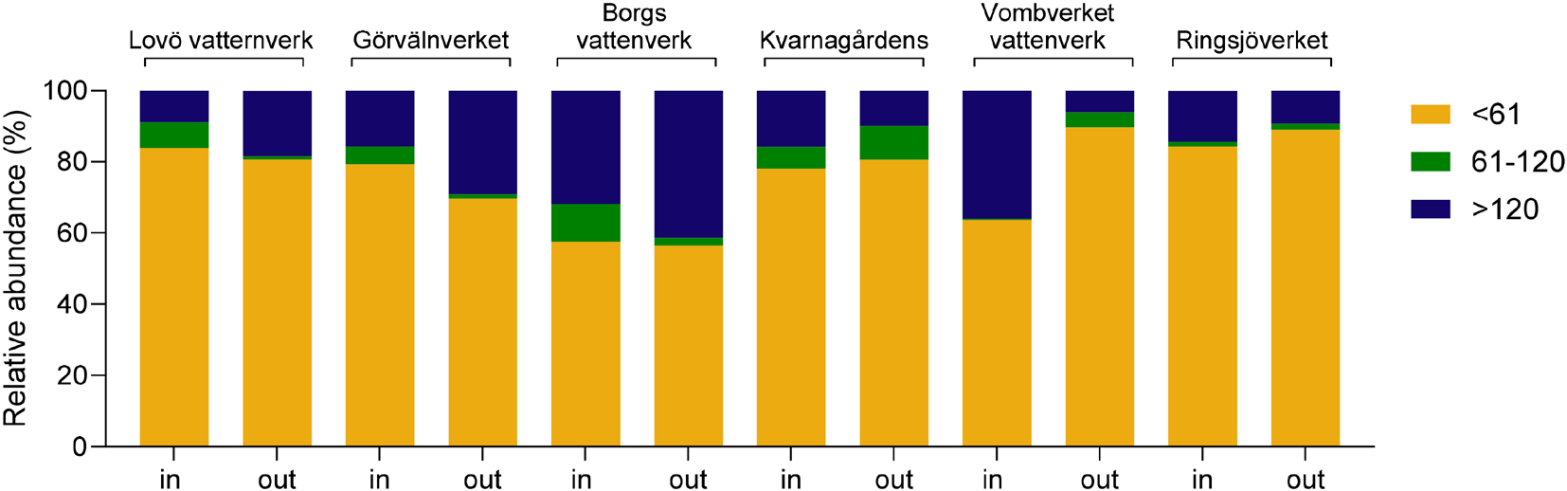
The percentage or relative abundance of the number of different viral contigs in raw water and drinking water samples based on virus size (nm) in diameter during the whole year.

There was a higher relative amount of the smaller viruses in the purified water compared to the raw water in three DWTPs, Kvarnagårdens vattenverk, Ringsjöverket and Vombverket (Figure 4). For Kvarnagårdens vattenverk, which is the only DWTP in this study that uses ultrafiltration, the viral contigs were reduced for all virus sizes, but the highest number of viral contigs in the drinking water were the smallest viruses (Table 1). For Vombverket, the most efficient reduction was for the larger viruses (Table 1). Viral contigs representing viruses of a size between 61 and 120 were effectively reduced to 2.15–3.68 log_10_ at Lovö vattenverk, Görvälnverket, Vombverket and Ringsjöverket (Table 1). Lovö vattenverk and Görvälnverket use very similar technologies, with a difference being that Görvälnverket uses carbon filtration instead of slow sand filtration (Figure 2). There was a mean increase in the relative amount of viral contigs representing large viruses in the drinking water from Görvälnverket and, to some extent, also at Borgs vattenverk (Table 1 and Table S5). The treatment of raw water to drinking water at all DWTPs, on average, significantly reduced contigs representing viruses larger than 60 nm more effectively than smaller viruses (*p* < 0.05). At Borgs vattenverk, which had the lowest amount of virus contigs in the raw water, the reduction of contigs homologous to viruses > 120 nm in size was less effective than at the other DWTPs (Table 1).

### 3.3 Presence of Viruses in the Waters in Relation to Virus‐Host

When the confirmed viral contigs were classified into three groups based on known hosts of the viruses, 48%–83% of the contigs from the raw water and 63%–85% of those from the drinking water samples could be classified into Group 1 (viruses infecting hosts which may be present in the treatment process; Table 2). The mean relative abundance of these contigs in the purified water samples was similar to or lower than that in the raw water samples for all DWTPs (Table S6). The most abundant viral contigs in this group were bacteriophages from 33 different virus families, followed by large viruses infecting algae or amoeba, and viruses found in marine environments (Table S4).

**FIGURE 5:**
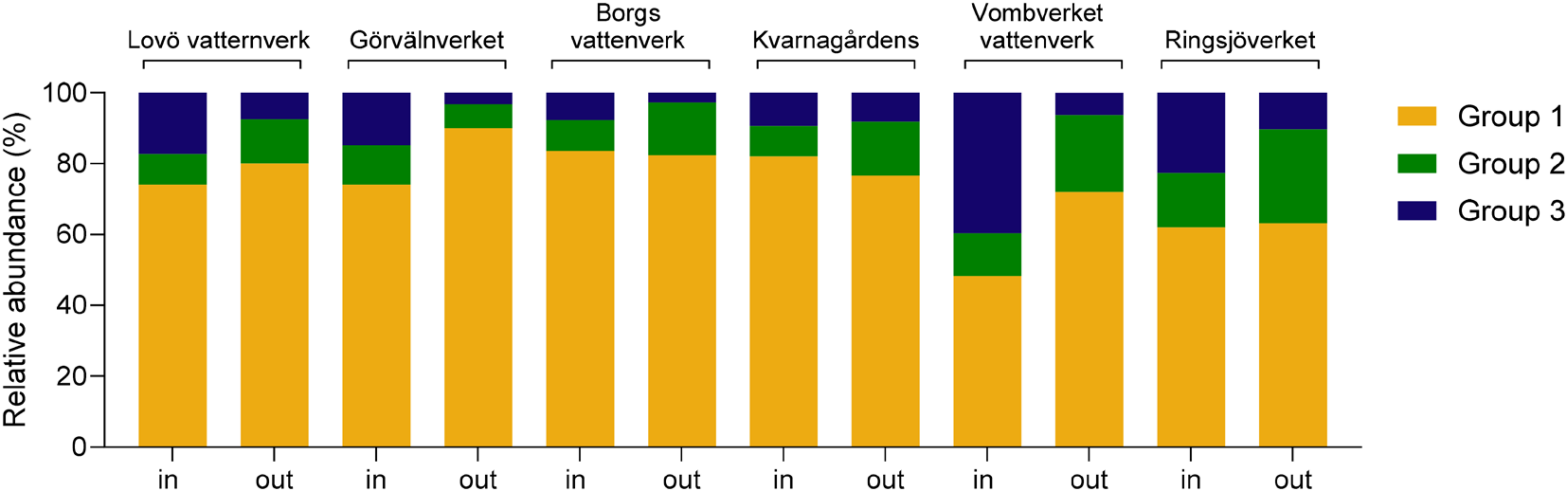
The percentage or relative abundance of viral contigs in all raw water and drinking water samples with regard to the type of virus‐host for each treatment plant for the whole year. Group 1 is viruses that may have their host present during the purification process, Group 2 is viruses with hosts probably not present during the purification process, and Group 3 is viruses with no host present during the purification process.

The mean prevalence of contigs was quite similar in all water samples for Group 2 (viruses infecting hosts unlikely to be present in the treatment process), ranging from 8.9% to 20.8% in the raw water and 6.4% to 26.6% in the treated water. All Group 2 contigs were homologous to sequences of bacteriophages infecting bacteria usually found in samples from vertebrates and plants. Some of these bacterial hosts are used as faecal indicators, such as *Escherichia coli* (Table 2). Although the total number of viral contigs was much lower in the treated water compared to the raw water, the proportional amount of contigs classified as Group 2 was higher or equal in the treated water compared to the raw water in all but one DWTP, Görvälnverket, where it was halved (Figure 5). At this DWTP, there was an increase in the influx of Group 2 sequences in May and September, when they were reduced by 4.4 log_10_ and 4.2 log_10_ during the purification process (Table S6).

In the third group (viruses infecting hosts not present in the treatment process), there were contigs homologous to viruses infecting invertebrates, vertebrates and plants. Of all contigs, 7.6%–39.2% in the raw water and 2.7%–10.3% in the treated water were homologous to Group 3 viruses (Figure 5). The percentage was lower or equal in samples of the treated water compared to those of raw water from the DWTPs (Figure 5). There was a high amount of Group 3 sequences in the raw water in Vombverket, especially between August and September, and it was caused by contigs homologous to viruses in the *Poxviridae* (infecting swine and rodents), *Orthoherpesviridae* (infecting ruminants, horses, swine, rodents and humans), and *Betaflexviridae* (infecting potatoes). There were also contigs homologous to bat viruses in the *Coronaviridae* family and human and ruminant viruses in the *Caliciviridae*, *Bornavirida*e and *Papillomaviridae* families.

The mean reduction of the viral contigs was 1 to 3 log_10_ in all DWTPs. The reduction of contigs homologous to sequences of viruses in Group 3, with no host in the purification process, was higher than that for bacteriophages for all DWTPs (Table 2). In Group 1, Lovö vattenverk, Görvälnverket and Kvarnagårdens vattenverk showed reductions where *p* values were nearly significant (*p* < 0.05) (Table 2).

### 3.4 Temporal Differences in Contigs Homologous to Viral Sequences at the Different DWTPs

**FIGURE 6:**
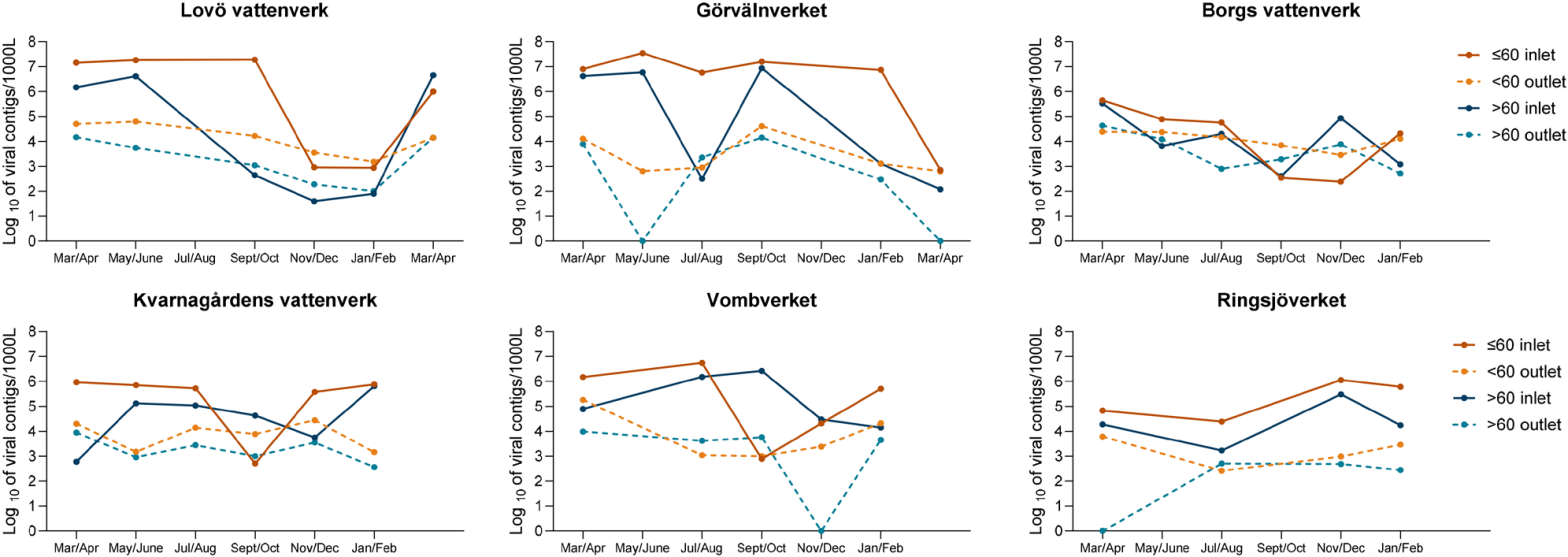
The number of contigs homologous to sequences of viruses (*y*‐axis) of different sizes in raw (inlet) and treated water (outlet) at the indicated sampling occasions at each DWTP.

**FIGURE 7:**
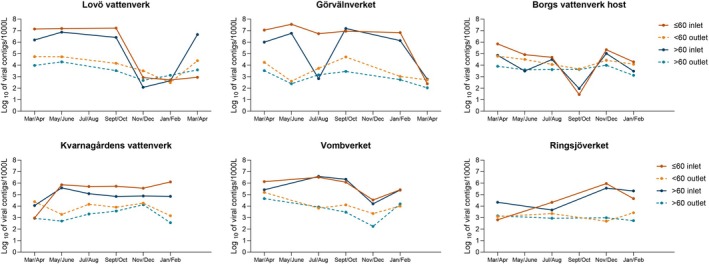
The number of contigs homologous to sequences (*y* axis) of viruses classified into three groups based on their hosts in raw (inlet) and treated water (outlet) at the indicated sampling occasions at each DWTP.

Contigs homologous to viruses of all categories tended to be more prevalent in the raw waters collected in March–July, followed by a decrease in November–December (Figures 6 and 7) in the three DWTPs with the highest number of contigs in the raw water samples (Lovö vattenverk, Görvälnverket and Vombverket). For Borgs vattenverk and Ringsjöverket, two plants with a lower number of viral contigs in the raw water samples, two peaks were observed between March–April and November–December. The number of contigs in the raw water was rather stable in the samples from Kvarnagårdens vattenverk during the whole year (Figures 6 and 7). The variation in the number of viral contigs in the purified drinking water followed that of the corresponding raw water from the same time point with a 1–3 log_10_ reduction. For water samples collected in May/June and September/October from Borgs vattenverk, there was an overlap of viral contigs representing viruses of different sizes and host types between raw water and drinking water (Figures 6, 7 and Tables S5–S6). At these time points, there was no reduction of the contigs with homologous sequences to larger viruses. These contigs represented mainly giant viruses belonging to the *Phycodnaviridae* family and Pandora viruses infecting algae and amoeba. There was also an increase in contigs homologous to viruses in the *Adenoviridae* family infecting birds. However, at these collection time points, there was at least a 1‐log_10_ reduction of contigs representing all Group 1 and Groups 2–3 viruses also at Borgs vattenverk (Table 2).

## 4 Discussion

The six DWTPs studied used five different combinations of purification barriers, none of which removed all viruses completely. Viral contigs were, however, reduced by 1–3 log_10_ after purification into drinking water. Although virus homologous sequences were detected in drinking water samples, the risk of being infected by drinking treated water is probably negligible as levels of human pathogenic viruses are very low (Saguti et al. 2022, 2023; Shirakawa et al. 2023). However, there might be a potential risk of infectious virus outbreaks with a high enough amount of viable infectious viruses in the raw water from human faeces or by animals infected with viruses with zoonotic potential, such as hepatitis E virus or other potential human pathogens (Benavent et al. 2023; Nemes et al. 2023; Turlewicz‐Podbielska et al. 2023; Chen et al. 2021). Outbreaks, though rare, have been known to happen, also here in Sweden (Larsson et al. 2014). This requires high capacity of the treatment technologies used at the DWTPs, as well as a warning system to detect viruses passing through the DWTP. Development of a relevant warning system depends on knowledge of which viruses are detected in drinking water from the DWTPs. Bacteriophages are used for monitoring water safety, and virus levels in drinking water distribution systems and as indicators of faecal contamination in water (Kelmer et al. 2023; Park et al. 2020). Knowledge is also needed about the viability of viruses in the treated water and the infectious dose of viruses that cause disease. Studying the viability of viruses is important, but more difficult as high levels of virus are needed in the water to allow for infectivity studies. In this study, we were only able to detect viral genomes; thus, the viruses we find may not be infectious.

The majority of viral contigs in all water samples in this study, both raw and treated water, belonged to viruses with hosts in aquatic environments. The most abundant contigs of this group were bacteriophages and giant viruses. They are widely distributed and abundant in water and specifically infect bacteria, algae or amoeba (Castledine and Buckling 2024). The results from this study indicated these viruses to be prevalent in the surface waters in Sweden that are used for drinking water production, which is consistent with the virome of surface water described in other studies (Lu et al. 2022; Cai et al. 2016; Ge et al. 2013; Rosario et al. 2009). Drinking water authorities in Sweden and the United States suggested using somatic coliphages as a faecal virus indicator (Vatten 2015; USEPA 2015), since they are abundant in drinking water sources. Their presence in treated water indicates a potential risk of contamination by human enteric viruses in the treated water (Singh et al. 2022). In addition to coliphages, emerging viruses such as crAssphage and pepper mild mottle virus (PMMoV) are gaining increasing attention as potential surrogates for human enteric viruses in evaluating drinking water treatment and faecal contamination (Shirasaki et al. 2020; Meuchi et al. 2023). However, their use is still under evaluation, and clear criteria for their reliability and applicability are lacking. Clear distinction of bacteriophages detected in drinking water sources based on host type in aquatic environments and prevalence can add value in the risk management of water contaminations, particularly during seasons of high pollution.

The most abundant contigs in the drinking water samples were homologous to small‐size viruses, 1–60 nm, which highlights the effectiveness of DWTPs in removing large‐size viruses and that it is important to take virus size into account when monitoring viruses in drinking water. Most of these viruses were bacteriophages, but invertebrate viruses and mammalian parvoviruses were also identified. The only DWTP in this study equipped with ultrafiltration with a membrane of pore size 20 nm, Kvarnagårdens vattenverk, showed a substantial reduction in the number of virus contigs, with lower levels of virus contigs larger than 60 nm in the drinking water compared to 1–60 nm as detected by NGS. This efficient removal of larger viruses by ultrafiltration combined with coagulation has also been described in other studies (Wang et al. 2020; Qiu et al. 2015). This result indicates that small‐size viruses, or non‐infectious viral genomes, may penetrate through the ultrafiltration membranes into the drinking water. Consistent with that, other studies have also detected small viruses in both raw and drinking water (Wang et al. 2020; Asami et al. 2016). Many human pathogenic enteric viruses are about 28–35 nm, with members of *Rotaviridae* and *Adenoviridae* being up to 80 nm in size (Bishop and Kirkwood 2008). Previously, the Swedish authority recommended a 100 nm pore size of the ultrafiltration membrane for drinking water production (Vatten 2015). Recently, a pore size of 40 nm has been recommended (Lidén 2020), which can still allow most human enteric viruses to pass through the filter. The use of ultrafiltration with small pore size membranes should be considered by DWTPs for water sources vulnerable to viral contaminations, especially those located close to big cities.

A study on freshwater planktonic viruses reported similar results regarding the temporal occurrence of bacteriophages as was identified in this study, with an increase in bacteriophage diversity in aquatic environments from late summer to December (Ge et al. 2013). The reason for a seasonal variation in bacteriophage abundance during the year might reflect a seasonal shift in host organisms in the water (Lopez‐Bueno et al. 2009). In contrast, the sequential increase in contigs homologous to viruses without hosts in water during the summer months can be explained by the presence of viruses infecting poultry/cattle/wild mammals/insects and from agricultural activities (Shen et al. 2024). In this study, two DWTPs used source water from Lake Mälaren near the Swedish capital Stockholm. Both DWTPs had the highest amount of virus contigs in their raw water samples, especially during spring (March–April) and autumn (September–October) weather. Virus concentration variation in the water samples from the lakes might be associated with the natural spring‐fall turnover, a phenomenon whereby a density gradient of water masses is formed due to solar radiation and drives a perpendicular turnover between warmer and cooler water regions in the lake (Malm 1995). Spring‐fall turnover has been associated with the accumulation of microorganisms due to a shift of nutrients and algae‐biomass blooming in the lakes (Kivilä et al. 2023). Furthermore, pollution from human activities, livestock and animals living in water source areas might also be sources of virus contamination.

This study showed virome variability in raw water samples collected from lakes, rivers, artificial groundwater and 20% groundwater used for drinking water production. The analysis was performed by NGS and classified into virus families. In addition, the effectiveness of six different DWTPs with different treatment methods in reducing the amount of virus and their fluctuation over the course of a year was assessed. These results were strengthened by the use of metagenomics techniques in identifying uncultivable viruses and providing preliminary data on the effectiveness of DWTPs in virus reduction. However, these studies are based on the analysis of NGS sequences, which have several weaknesses and can only give indications of the number of viruses present in the samples (Boers et al. 2019). One of the main limitations of this metagenomic technique is the sensitivity of the amplification of sequences by PCR before library preparation for NGS, with the risk of viral genomes not being amplified with the random primers used in the PCR. Viral genomes present in the water may be missed during this step. It has repeatedly been shown to be less sensitive than qPCR and underestimation of viral diversity (Cantalupo et al. 2011; Wang et al. 2018). Also, differences in read depth will affect the number of virus contigs assembled when comparing contigs between samples. Another drawback is an overestimation of the number of identified viruses during blasting by giving false‐positive hits (Boers et al. 2019). In this study, two sets of databases were used to reduce false positive viral reads. First, a database with viral sequences was searched by the BLASTn database in GenBank. Then the results were used for a search in the Kraken2 database in the European Galaxy. This data was also analysed using the geNomad database, showing a similar number of viral sequences compared to the BLASTn database. This limitation in the identification of viruses in the water samples may have reduced the true number of viruses that were present. This may also have caused the loss of smaller viral genomes and an overestimation of the concentration of the larger viral genomes. Also, Lovö vattenverk and Görvälnverket, due to clogging of the Nano‐ceram filters, had to send 20 L of raw water for purification with Nano‐ceram filters at the lab. This may have affected the amounts of viruses detected in the raw water, though both of these DWTPs were able to detect a comparative number of viral reads in the raw water samples. The intent of the study was not to identify all viruses or provide exact quantification of the viruses, but to identify which well‐characterised viruses were present in higher concentrations in all of the DWTPs to be compared over the study period to detect if there were differences between DWTP treatment techniques or raw water sources and/or temporal differences, as well as detect viruses not previously known to be present in higher amounts in raw water samples. Despite these drawbacks, this study lays the foundation for a better understanding of the virome in different waters and its temporal variation. Based on these results, we can investigate which specific viruses should be followed up to detect increased viral load in the water samples. Viruses present in many water sources that cannot be amplified in the DWTP due to infection of a host in the water system, and detectable throughout the system, may be used to in more detail determine the efficiency of purification steps in DWTPs with methods that are more suitable for detailed quantification. Using viruses of different sizes would provide information of variable efficiency dependent on virus size in filtration processes. The study has also specifically shown the additional knowledge required to be able to follow the treatment techniques in different DWTPs and detect if the virus load is increasing in the waters. Further studies with more sensitive molecular techniques are needed to determine the exact efficiency of water treatment barriers against virus load detected in DWTPs.

## 5 Conclusions

This study used metagenomic analysis to assess the virome in raw and drinking water samples from six Swedish DWTPs equipped with different barriers. The majority of viruses detected with metagenomics were bacteriophages. Many were small, comparable in size to most human pathogenic enteric viruses. Other viral genomes were homologous to viruses infecting plants, invertebrates, vertebrates, mammals and giant viruses infecting amoeba or algae. Very few viruses detected were human pathogens. Viruses were detected in both raw and drinking water, and viral contigs were reduced by 1–3 log_10_. The concentration of viruses in drinking water varied throughout the year and followed the same pattern as that in raw water for most DWTPs. Virus removal by ultrafiltration increased proportionally with virus size with more virus contigs of 1–60 nm in the treated water. Other barriers appeared to be more effective at removing smaller viruses. These results underscore the importance of monitoring small viruses, especially during times of the year with a high potential for viral contamination of the source water, to ensure drinking water quality.

## Author Contributions

**Fredy Saguti:** investigation, formal analysis, writing – original draft, visualization. **Hao Wang:** investigation, writing – review and editing. **Marianela Patzi Churqui:** visualization, writing – review and editing. **Timur Tunovic:** investigation, writing – review and editing. **Linda Holmer:** funding acquisition, writing – review and editing, investigation. **Ämma Pettersson:** funding acquisition, investigation, writing – review and editing. **Caroline Schleich:** investigation, funding acquisition, writing – review and editing. **Britt‐Marie Pott:** investigation, funding acquisition, writing – review and editing. **Olof Bergstedt:** writing – review and editing. **Kristina Nyström:** conceptualization, formal analysis, project administration, writing – review and editing, funding acquisition. **Heléne Norder:** conceptualization, formal analysis, funding acquisition, project administration, supervision, visualization, writing – review and editing.

## Conflicts of Interest

The authors declare no conflicts of interest.

## Supporting information


**Data S1:** emi470222‐sup‐0001‐Supinfo.docx.

## Data Availability

The data that supports the findings of this study is available in Supporting Information of this article.

## References

[emi470222-bib-0001] Albinana‐Gimenez, N. , M. P. Miagostovich , B. Calgua , J. M. Huguet , L. Matia , and R. Girones . 2009. “Analysis of Adenoviruses and Polyomaviruses Quantified by qPCR as Indicators of Water Quality in Source and Drinking‐Water Treatment Plants.” Water Research 43: 2011–2019. 10.1016/j.watres.2009.01.025. 19230949

[emi470222-bib-0002] Altschul, S. F. , W. Gish , W. Miller , E. W. Myers , and D. J. Lipman . 1990. “Basic Local Alignment Search Tool.” Journal of Molecular Biology 215: 403–410. 10.1016/S0022-2836(05)80360-2. 2231712

[emi470222-bib-0003] Asami, T. , H. Katayama , J. R. Torrey , C. Visvanathan , and H. Furumai . 2016. “Evaluation of Virus Removal Efficiency of Coagulation‐Sedimentation and Rapid Sand Filtration Processes in a Drinking Water Treatment Plant in Bangkok, Thailand.” Water Research 101: 84–94. 10.1016/j.watres.2016.05.012. 27258619

[emi470222-bib-0004] Benavent, S. , S. Carlos , and G. Reina . 2023. “Rocahepevirus Ratti as an Emerging Cause of Acute Hepatitis Worldwide.” Microorganisms 11: 2996. 10.3390/microorganisms11122996. 38138140 PMC10745784

[emi470222-bib-0005] Bibby, K. , K. Crank , J. Greaves , et al. 2019. “Metagenomics and the Development of Viral Water Quality Tools.” npj Clean Water 2: 9–13.

[emi470222-bib-0006] Bishop, R. F. , and C. D. Kirkwood . 2008. “Enteric Viruses.” In Encyclopedia of Virology, 116–123. Science Direct, Elsevier. 10.1016/B978-012374410-4.00386-1.

[emi470222-bib-0007] Boers, S. A. , R. Jansen , and J. P. Hays . 2019. “Understanding and Overcoming the Pitfalls and Biases of Next‐Generation Sequencing (NGS) Methods for Use in the Routine Clinical Microbiological Diagnostic Laboratory.” European Journal of Clinical Microbiology & Infectious Diseases: Official Publication of the European Society of Clinical Microbiology 38: 1059–1070. 10.1007/s10096-019-03520-3. 30834996 PMC6520317

[emi470222-bib-0008] Böttiger, M. , and E. Herrström . 1992. “Isolation of Polioviruses From Sewage and Their Characteristics: Experience Over Two Decades in Sweden.” Scandinavian Journal of Infectious Diseases 24: 151–155. 10.3109/00365549209052605. 1322558

[emi470222-bib-0009] Cai, L. , R. Zhang , Y. He , X. Feng , and N. Jiao . 2016. “Metagenomic Analysis of Virioplankton of the Subtropical Jiulong River Estuary, China.” Viruses 8: 35. 10.3390/v8020035. 26848678 PMC4776190

[emi470222-bib-0010] Cantalupo, P. G. , B. Calgua , G. Zhao , et al. 2011. “Raw Sewage Harbors Diverse Viral Populations.” MBio 2: 1–11. 10.1128/mBio.00180-11. PMC3187576 21972239

[emi470222-bib-0011] Carol, M. , V. Guadalupe‐Fernández , C. Rius , et al. 2021. “A Waterborne Gastroenteritis Outbreak Caused by a GII Norovirus in a Holiday Camp in Catalonia (Spain), 2017.” Viruses 13: 1792. 10.3390/v13091792. 34578373 PMC8473012

[emi470222-bib-0012] Castledine, M. , and A. Buckling . 2024. “Critically Evaluating the Relative Importance of Phage in Shaping Microbial Community Composition.” Trends in Microbiology 32: 957–969. 10.1016/j.tim.2024.02.014. 38604881

[emi470222-bib-0013] Chalmers, R. M. 2012. “Waterborne Outbreaks of Cryptosporidiosis.” Annali dell'Istituto Superiore di Sanità 48: 429–446. 10.4415/ann_12_04_10. 23247139

[emi470222-bib-0014] Chen, L. , Y. Deng , S. Dong , et al. 2021. “The Occurrence and Control of Waterborne Viruses in Drinking Water Treatment: A Review.” Chemosphere 281: 130728. 10.1016/j.chemosphere.2021.130728. 34010719 PMC8084847

[emi470222-bib-0015] Chen, Y. J. , N. X. Cao , R. H. Xie , et al. 2016. “Epidemiological Investigation of a Tap Water‐Mediated Hepatitis E Virus Genotype 4 Outbreak in Zhejiang Province, China.” Epidemiology and Infection 144: 3387–3399. 10.1017/S0950268816001898. 27546066 PMC9150197

[emi470222-bib-0016] Delpla, I. , A. V. Jung , E. Baures , M. Clement , and O. Thomas . 2009. “Impacts of Climate Change on Surface Water Quality in Relation to Drinking Water Production.” Environment International 35: 1225–1233. 10.1016/j.envint.2009.07.001. 19640587

[emi470222-bib-0017] Espinosa, A. C. , M. Mazari‐Hiriart , R. Espinosa , L. Maruri‐Avidal , E. Méndez , and C. F. Arias . 2008. “Infectivity and Genome Persistence of Rotavirus and Astrovirus in Groundwater and Surface Water.” Water Research 42: 2618–2628. 10.1016/j.watres.2008.01.018. 18291437

[emi470222-bib-0018] Figas, A. , M. Wieczorek , B. Litwińska , and W. Gut . 2017. “Detection of Polioviruses in Sewage Using Cell Culture and Molecular Methods.” Polish Journal of Microbiology 65: 479–483. 10.5604/17331331.1227676. 28735334

[emi470222-bib-0019] Ge, X. , Y. Wu , M. Wang , et al. 2013. “Viral Metagenomics Analysis of Planktonic Viruses in East Lake, Wuhan, China.” Virologica Sinica 28: 280–290. 10.1007/s12250-013-3365-y. 24132758 PMC8208453

[emi470222-bib-0020] Kauppinen, A. , T. Pitkänen , H. al‐Hello , et al. 2019. “Two Drinking Water Outbreaks Caused by Wastewater Intrusion Including Sapovirus in Finland.” International Journal of Environmental Research and Public Health 16: 4376. 10.3390/ijerph16224376. 31717479 PMC6888097

[emi470222-bib-0053] Kelliher, J. M. , Y. Xu , M. C. Flynn , et al. 2024. “Standardized and Accessible Multi‐Omics Bioinformatics Workflows Through the NMDC EDGE Resource.” Computational and Structural Biotechnology Journal 23: 3575–3583. 39963423 10.1016/j.csbj.2024.09.018 PMC11832004

[emi470222-bib-0021] Kelmer, G. A. R. , E. R. Ramos , and E. H. O. Dias . 2023. “Coliphages as Viral Indicators in Municipal Wastewater: A Comparison Between the ISO and the USEPA Methods Based on a Systematic Literature Review.” Water Research 230: 119579. 10.1016/j.watres.2023.119579. 36640612

[emi470222-bib-0022] Kivilä, E. H. , V. Prėskienis , N. Gaudreault , C. Girard , and M. Rautio . 2023. “Variability in Lake Bacterial Growth and Primary Production Under Lake Ice: Evidence From Early Winter to Spring Melt.” Limnology and Oceanography 68: 2603–2616.

[emi470222-bib-0023] Kotwal, G. , and J. L. Cannon . 2014. “Environmental Persistence and Transfer of Enteric Viruses.” Current Opinion in Virology 4: 37–43. 10.1016/j.coviro.2013.12.003. 24413147

[emi470222-bib-0024] Larsson, C. , Y. Andersson , G. Allestam , et al. 2014. “Epidemiology and Estimated Costs of a Large Waterborne Outbreak of Norovirus Infection in Sweden.” Epidemiology and Infection 142: 592–600. 10.1017/S0950268813001209. 23714107 PMC9151097

[emi470222-bib-0025] Lefkowitz, E. J. , D. M. Dempsey , R. C. Hendrickson , R. J. Orton , S. G. Siddell , and D. B. Smith . 2018. “Virus Taxonomy: The Database of the International Committee on Taxonomy of Viruses (ICTV).” Nucleic Acids Research 46: D708–D717. 10.1093/nar/gkx932. 29040670 PMC5753373

[emi470222-bib-0026] Lidén, A. 2020. “Membranfiltrering för dricksvattenberedningen kunskaps sammanställning Svenskt Vatten AB.”

[emi470222-bib-0027] Lopez‐Bueno, A. , J. Tamames , D. Velázquez , et al. 2009. “High Diversity of the Viral Community From an Antarctic Lake.” Science 326: 858–861. 10.1126/science.1179287. 19892985

[emi470222-bib-0028] Lu, J. , S. Yang , X. Zhang , et al. 2022. “Metagenomic Analysis of Viral Community in the Yangtze River Expands Known Eukaryotic and Prokaryotic Virus Diversity in Freshwater.” Virologica Sinica 37: 60–69. 10.1016/j.virs.2022.01.003. 35234628 PMC8922420

[emi470222-bib-0029] Malm, J. 1995. “Spring Circulation Associated With the Thermal Bar in Large Temperate Lakes.” Nordic Hydrology 26: 331–358.

[emi470222-bib-0030] Meuchi, Y. , M. Nakada , K. Kuroda , S. Hanamoto , and A. Hata . 2023. “Applicability of F‐Specific Bacteriophage Subgroups, PMMoV and crAssphage as Indicators of Source Specific Fecal Contamination and Viral Inactivation in Rivers in Japan.” PLoS One 18: e0288454. 37450468 10.1371/journal.pone.0288454 PMC10348522

[emi470222-bib-0031] Moreira, N. A. , and M. Bondelind . 2017. “Safe Drinking Water and Waterborne Outbreaks.” Journal of Water and Health 15: 83–96. 10.2166/wh.2016.103. 28151442

[emi470222-bib-0032] Nemes, K. , S. Persson , and M. Simonsson . 2023. “Hepatitis A Virus and Hepatitis E Virus as Food‐ and Waterborne Pathogens‐Transmission Routes and Methods for Detection in Food.” Viruses 15: 1725. 10.3390/v15081725. 37632066 PMC10457876

[emi470222-bib-0033] Park, G. W. , T. F. F. Ng , A. L. Freeland , et al. 2020. “CrAssphage as a Novel Tool to Detect Human Fecal Contamination on Environmental Surfaces and Hands.” Emerging Infectious Diseases 26: 1731–1739. 10.3201/eid2608.200346. 32511090 PMC7392416

[emi470222-bib-0034] Pinon, A. , and M. Vialette . 2018. “Survival of Viruses in Water.” Intervirology 61: 214–222. 10.1159/000484899. 29316545

[emi470222-bib-0035] Qiu, Y. , B. E. Lee , N. Neumann , et al. 2015. “Assessment of Human Virus Removal During Municipal Wastewater Treatment in Edmonton, Canada.” Journal of Applied Microbiology 119: 1729–1739. 10.1111/jam.12971. 26473649

[emi470222-bib-0036] Rosario, K. , C. Nilsson , Y. W. Lim , Y. Ruan , and M. Breitbart . 2009. “Metagenomic Analysis of Viruses in Reclaimed Water.” Environmental Microbiology 11: 2806–2820. 10.1111/j.1462-2920.2009.01964.x. 19555373

[emi470222-bib-0037] Saguti, F. , M. P. Churqui , I. Kjellberg , et al. 2022. “The UV Dose Used for Disinfection of Drinking Water in Sweden Inadequately Inactivates Enteric Virus With Double‐Stranded Genomes.” International Journal of Environmental Research and Public Health 19: 8669. 10.3390/ijerph19148669. 35886521 PMC9316100

[emi470222-bib-0038] Saguti, F. , I. Kjellberg , M. P. Churqui , et al. 2023. “The Virucidal Effect of the Chlorination of Water at the Initial Phase of Disinfection May be Underestimated if Contact Time Calculations Are Used.” Pathogens 12: 1216. 10.3390/pathogens12101216. 37887732 PMC10609707

[emi470222-bib-0039] Saguti, F. , E. Magnil , L. Enache , et al. 2021. “Surveillance of Wastewater Revealed Peaks of SARS‐CoV‐2 Preceding Those of Hospitalized Patients With COVID‐19.” Water Research 189: 116620. 10.1016/j.watres.2020.116620. 33212338 PMC7654368

[emi470222-bib-0040] Seitz, S. R. , J. S. Leon , K. J. Schwab , et al. 2011. “Norovirus Infectivity in Humans and Persistence in Water.” Applied and Environmental Microbiology 77: 6884–6888. 10.1128/aem.05806-11. 21856841 PMC3187119

[emi470222-bib-0041] Shen, L. , Z. Zhang , R. Wang , S. Wu , Y. Wang , and S. Fu . 2024. “Metatranscriptomic Data Mining Together With Microfluidic Card Uncovered the Potential Pathogens and Seasonal RNA Viral Ecology in a Drinking Water Source.” Journal of Applied Microbiology 135: 1–14. 10.1093/jambio/lxad310. 38130237

[emi470222-bib-0042] Shirakawa, D. , N. Shirasaki , Q. Hu , et al. 2023. “Investigation of Removal and Inactivation Efficiencies of Human Sapovirus in Drinking Water Treatment Processes by Applying an In Vitro Cell‐Culture System.” Water Research 236: 119951. 10.1016/j.watres.2023.119951. 37060876

[emi470222-bib-0043] Shirasaki, N. , T. Matsushita , Y. Matsui , and S. Koriki . 2020. “Suitability of Pepper Mild Mottle Virus as a Human Enteric Virus Surrogate for Assessing the Efficacy of Thermal or Free‐Chlorine Disinfection Processes by Using Infectivity Assays and Enhanced Viability PCR.” Water Research 186: 116409. 32942179 10.1016/j.watres.2020.116409

[emi470222-bib-0044] Singh, S. , R. Pitchers , and F. Hassard . 2022. “Coliphages as Viral Indicators of Sanitary Significance for Drinking Water.” Frontiers in Microbiology 13: 941532. 10.3389/fmicb.2022.941532. 35958148 PMC9362991

[emi470222-bib-0045] Sylvestre, É. , M. Prévost , J. B. Burnet , et al. 2021. “Demonstrating the Reduction of Enteric Viruses by Drinking Water Treatment During Snowmelt Episodes in Urban Areas.” Water Research X 11: 100091. 10.1016/j.wroa.2021.100091. 33598650 PMC7868990

[emi470222-bib-0046] Turlewicz‐Podbielska, H. , A. Augustyniak , J. Wojciechowski , and M. Pomorska‐Mól . 2023. “Hepatitis E Virus in Livestock‐Update on Its Epidemiology and Risk of Infection to Humans.” Animals 13: 1–24. 10.3390/ani13203239. PMC10603682 37893962

[emi470222-bib-0047] USEPA . 2015. “Review of Coliphages as Possible Indicators of Fecal Contamination for Ambient Water Quality.”

[emi470222-bib-0048] Vatten, S. 2015. “Introduktion till mikrobiologisk barriaranalys.”

[emi470222-bib-0049] Wang, H. , I. Kjellberg , P. Sikora , et al. 2020. “Hepatitis E Virus Genotype 3 Strains and a Plethora of Other Viruses Detected in Raw and Still in Tap Water.” Water Research 168: 115141. 10.1016/j.watres.2019.115141. 31590036

[emi470222-bib-0050] Wang, H. , P. Sikora , C. Rutgersson , et al. 2018. “Differential Removal of Human Pathogenic Viruses From Sewage by Conventional and Ozone Treatments.” International Journal of Hygiene and Environmental Health 221: 479–488. 10.1016/j.ijheh.2018.01.012. 29402695 PMC7106402

[emi470222-bib-0052] Yates, M. V. , C. P. Gerba , and L. M. Kelley . 1985. “Virus Persistence in Groundwater.” Applied and Environmental Microbiology 49: 778–781. 10.1128/aem.49.4.778-781.1985. 4004211 PMC238444

