# Supplementary material for: Variations of the Virome in Raw and Treated Water: A One‐Year Follow‐Up at Six Different Drinking Water Treatment Plants
Source: Environ Microbiol Rep. 2025 Oct 28;17(6):e70222. doi: 10.1111/1758-2229.70222 (PMC12566884; doi:10.1111/1758-2229.70222)
Supplement: Supplementary file 1 — Data S1: emi470222‐sup‐0001‐Supinfo.docx. [file EMI4-17-e70222-s001.docx]

| **DWTP** | **Treatment barriers** |
| --- | --- |
| Lovö vattenverk | Microsieves, Flocculation, Sedimentation, Rapid sand filtration, slow sand filtration,  UV light and disinfection (monochloramine). |
| Görvälnverket | Flocculation, Sedimentation, Rapid sand filtration, Carbon filtration,  UV light and Disinfection |
| Borgs vattenverk | Flocculation, Sedimentation, Rapid carbon filtration, Slow sand filtration,  UV light and disinfection (monochloramine) |
| Kvarnagårdens  vattenverk | Rapid sand filtration,  Ultrafiltration combined with coagulation, UV light and disinfection |
|  |  |
| Vombverket | Micro-sieves, Infiltration, Aeration, Overflow mixing, Rapid Sand filtration, and Disinfection (monochloramine). |
| Ringsjöverket | Flocculation, Sedimentation, Rapid Sand filtration, Slow sand filtration, UV light and Disinfection. |

**Table S1,** DWTPs participating in this study and their treatment barriers

**Table S2.** Amount of raw water either filtered through Nanoceram filters at the six DWTPs or sent to Gothenburg for filtering (20 L) and amount of drinking water filtered through Nanoceram filters at the six DWTPs, at each collection time point.

| **DWTP** | **Water sample** | **2021** | | | | | | | | | | **2022** | | | | | |
| --- | --- | --- | --- | --- | --- | --- | --- | --- | --- | --- | --- | --- | --- | --- | --- | --- | --- |
|  |  | **Spring** | | | **Summer** | | | **Autumn** | | | **Winter** | | | | **Spring** | | |
|  |  | **March** | **April** | **May** | **June** | **July** | **August** | **September** | **October** | **November** | **December** | | **January** | **February** | **March** | **April** |  |
| Lovö vattenverk | Raw Water (L) |  | 20 |  | 20 |  |  |  | 20 |  | 20 | |  | 20 |  | 20 |  |
|  | Drinking Water (L) |  | 6,800 |  | 7,000 |  |  |  | 6,500 |  | 8,400 | |  | 7,855 |  | 6,600 |  |
| Görvälnverket | Raw Water (L) | 20 |  | 20 |  | 20 |  | 20 |  |  |  | | 20 |  | 20 |  |  |
|  | Drinking Water (L) | 16,560 |  | 19,872 |  | 8,820 |  | 5,829 |  |  |  | | 16,226 |  | 30,164 |  |  |
| Borgs vattenverk | Raw Water (L) |  | 370 |  | 7,498 |  | 1,444 |  | 2,000 |  | 580 | |  | 1,326 |  |  |  |
|  | Drinking Water (L) |  | 6,647 |  | 6,923 |  | 6,032 |  | 7,550 |  | 5,014 | |  | 6,121 |  |  |  |
| Kvarnagårdens  vattenverk | Raw Water (L) | 150 |  | 147 |  | 185 |  | 150 |  | 156 |  | | 153 |  |  |  |  |
|  | Drinking Water (L) | 9,360 |  | 9,530 |  | 9,245 |  | 9,740 |  | 9,930 |  | | 8,730 |  |  |  |  |
| Vombverket | Raw Water (L) |  | 255 |  | 7,228 |  | 44 |  | 80 |  | 349 | |  | 454 |  |  |  |
|  | Drinking Water (L) |  | 3,325 |  | 11,162 |  | 7,391 |  | 3,988 |  | 4,605 | |  | 4,262 |  |  |  |
| Ringsjöverket | Raw Water (L) | 7,085 |  | 306 |  | 7,119 |  | 302 |  | 307 |  | | 181 |  |  |  |  |
|  | Drinking Water (L) | 8,356 |  | 8,103 |  | 12,472 |  | 8,812 |  | 6,535 |  | | 5,766 |  |  |  |  |

**Table S3.** Number of reads, >500 nucleotide contigs, number of viral contigs, and number of confirmed viruses by both Kraken2 and Blastn databases for raw water (Inlet) and drinking water (Outlet) samples for each DWTP.

| **DWTPs** | **Reads/L water** | **2021** | | | | | | | | | | | **2022** | | | |
| --- | --- | --- | --- | --- | --- | --- | --- | --- | --- | --- | --- | --- | --- | --- | --- | --- |
|  |  | **March/April** | | **May/June** | | **July/August** | | **September/October** | | **November/December** | | **January/February** | | | **March/April** | |
| Lovö  vattenverk | Type of water sample | Inlet | Outlet | Inlet | Outlet | Inlet | Outlet | Inlet | Outlet | Inlet | Outlet | Inlet | | Outlet | Inlet | Outlet |
|  | Number reads | 2.32E+08 | 4.02E+07 | 1.49E+07 |  |  | 4.83E+07 | 3.25E+07 | 2.50E+07 | 1.57E+08 | 2.07E+08 | 4.65E+07 | | 1.98E+08 | 6.93E+07 | 2.32E+08 |
|  | Number reads/1000L | 3,28E+12 | 2,73E+10 | 1,61E+12 | 1,70E+09 |  |  | 1,93E+12 | 4,00E+09 | 9,98E+11 | 1,50E+10 | 8,28E+12 | | 4,74E+09 | 7,91E+12 | 8,40E+09 |
|  | >500 nucleotide contigs/1000L | 2,17E+09 | 9,76E+06 | 4,95E+09 | 5,51E+06 | Nil | Nil | 2,02E+09 | 5,15E+06 | 9,05E+08 | 2,42E+07 | 8,11E+08 | | 48 500 | 2,10E+09 | 5,94E+06 |
|  | Number confirmed viral contigs /1000L | 1,59E+07 | 65 059 | 2,28E+07 | 72 457 | Nil | Nil | 1,95E+07 | 17 846 | 960 000 | 3 714 | 960 000 | | 1 630 | 5,64E+06 | 28 242 |
|  | Confirmed viruses by Blastn and kraken2 | 29 | 28 | 39 | 47 | Nil | Nil | 25 | 25 | 9 | 8 | 13 | | 8 | 14 | 24 |
| Görväln-verket | Number reads | 4.12E+07 | 6.83E+07 | 5.14E+07 | 2.71E+07 | 3.11E+07 | 2.21E+07 | 3.34E+07 | 2.63E+07 | 1.46E+07 | 4.09E+07 | 9.01E+07 | | 7.10E+07 |  |  |
|  | Number reads/1000L | 1,65E+12 | 6,24E+09 | 2,06E+12 | 1,09E+09 | 1,24E+12 | 2,00E+09 | 1,34E+12 | 3,61E+09 | Nil | Nil | 5,85E+11 | | 2,02E+09 | 3,61E+12 | 1,88E+09 |
|  | >500 nucleotide contigs/1000L | 2,64E+09 | 6,03E+06 | 4,09E+09 | 1,18E+06 | 2,14E+09 | 3,25E+06 | 1,46E+09 | 4,66E+06 | Nil | Nil | 8,59E+08 | | 464 489 | 5,56E+08 | 500 995 |
|  | Number confirmed viral contigs /1000L | 1,23E+07 | 20 725 | 4,12E+07 | 644 | 6,12E+06 | 11 338 | 2,44E+07 | 55 035 | Nil | Nil | 8,72E+06 | | 1 578 | 840 000 | 637 |
|  | Confirmed viruses by Blastn and kraken2 | 32 | 45 | 69 | 4 | 24 | 8 | 33 | 28 | Nil | Nil | 37 | | 32 | 10 | 24 |
| Borgs  vattenverk | Number reads | 2.14E+07 | 3.23E+07 | 3.51E+07 | 4.30E+07 | 2.42E+07 | 3.03E+07 | 2.28E+07 | 3.34E+07 | 2.69E+07 | 3.42E+07 | 2.95E+07 | | 2.27E+07 |  |  |
|  | Number reads/1000L | 4,63E+10 | 3,88E+09 | 3,75E+09 | 4,97E+09 | 1,34E+10 | 4,02E+09 | 9,12E+09 | 3,54E+09 | 3,70E+10 | 5,46E+09 | 1,78E+10 | | 2,96E+09 |  |  |
|  | >500 nucleotide contigs/1000L | 5,81E+07 | 3,43E+06 | 6,13E+06 | 1,29E+07 | 3,14E+07 | 3,98E+06 | 1,13E+07 | 2,68E+06 | 3,52E+07 | 5,03E+06 | 1,32E+07 | | 6,58E+06 |  |  |
|  | Number confirmed viral contigs /1000L | 783 113 | 67 880 | 94 745 | 35 938 | 79 778 | 15 650 | 37 200 | 8 901 | 325 517 | 36 378 | 22 323 | | 13 332 |  |  |
|  | Confirmed viruses by Blastn and kraken2 | 27 | 34 | 35 | 42 | 27 | 24 | 23 | 23 | 22 | 22 | 12 | | 15 |  |  |
| Kvarnagård-ens  vattenverk | Number reads | 6.87E+07 | 6.09E+07 | 1.33E+08 | 1.24E+08 | 3.03E+07 | 8.55E+07 | 2.39E+07 | 5.28E+07 | 1.67E+07 | 1.12E+07 | 6.82E+07 | | 7.93E+07 |  |  |
|  | Number reads/1000L | 3,66E+11 | 5,20E+09 | 7,23E+11 | 1,04E+10 | 1,31E+11 | 7,40E+09 | 1,27E+11 | 4,34E+09 | 8,55E+10 | 9,02E+08 | 3,56E+11 | | 7,27E+09 |  |  |
|  | >500 nucleotide contigs/1000L | 2,43E+08 | 4,14E+06 | 2,63E+08 | 311 354 | 1,95E+08 | 1,20E+06 | 2,20E+08 | 1,36E+06 | 9,81E+07 | 1,07E+06 | 2,57E+08 | | 279 771 |  |  |
|  | Number confirmed viral contigs /1000L | 1,55E+06 | 29 145 | 1,11E+06 | 16 701 | 635 676 | 16 701 | 602 667 | 11 828 | 435 897 | 31 259 | 1,41E+06 | | 1 833 |  |  |
|  | Number confirmed viral contigs /1000L | 31 | 33 | 27 | 14 | 28 | 26 | 21 | 24 | 14 | 24 | 38 | | 11 |  |  |
| Vombverket | Number reads | 3.61E+07 | 3.36E+07 | 2.94E+07 | 1.94E+07 | 5.55E+07 | 6.85E+07 | 2.74E+07 | 2.39E+07 | 1.74E+07 | 1.10E+07 | 6.78E+07 | | 7.61E+07 |  |  |
|  | Number reads/1000L | 1,13E+11 | 8,09E+09 | 3,25E+09 | 1,39E+09 | 1,01E+12 | 7,41E+09 | 2,74E+11 | 4,79E+09 | 3,98E+10 | 1,90E+11 | 1,19E+11 | | 1,43E+10 |  |  |
|  | >500 nucleotide contigs/1000L | 8,57E+07 | 3,45E+07 | 228 224 | 1,81E+06 | 5,01E+08 | 2,71E+06 | 3,78E+08 | 6,90E+06 | 1,41E+07 | 3,83E+06 | 5,66E+07 | | 1,65E+06 |  |  |
|  | Number confirmed viral contigs /1000L | 1,62E+06 | 200 421 | 228 | 1 813 | 7,05E+06 | 15 261 | 3,54E+06 | 15 848 | 50 459 | 2 432 | 518 062 | | 25 716 |  |  |
|  | Confirmed viruses by Blastn and kraken2 | 30 | 32 | 1 | 17 | 24 | 18 | 25 | 16 | 11 | 6 | 35 | | 18 |  |  |
| Ringsjöverket | Number reads | 1.95E+07 | 6.86E+07 |  | 7.44E+07 | 5.64E+07 | 2.23E+07 | 3.91E+07 | 1.93E+07 | 3.04E+07 | 4.75E+07 | 8.80E+07 | | 7.70E+07 |  |  |
|  | Number reads/1000L | 2,21E+09 | 6,57E+09 | - | 7,35E+09 | 6,34E+09 | 1,43E+09 | 1,04E+11 | 1,75E+09 | 7,93E+10 | 5,82E+09 | 3,89E+11 | | 1,07E+10 |  |  |
|  | >500 nucleotide contigs/1000L | 7,95E+06 | 385 926 | - | 4,44E+06 | 2,31E+06 | 1,38E+06 | 7,92E+07 | 2,51E+06 | 1,27E+08 | 271 767 | 1,52E+08 | | 4,55E+06 |  |  |
|  | Number confirmed viral contigs /1000L | 86 267 | 2 681 | - | 85 | 26 071 | 3 143 | 5 081 | 418 | 1,45E+06 | 1 469 | 666 667 | | 3 191 |  |  |
|  | Confirmed viruses by Blastn and kraken2 | 43 | 6 | - | 30 | 33 | 12 | 56 | 34 | 40 | 8 | 23 | | 8 |  |  |

Note: Nil= water samples could not collected on the due date, instead water samples were collected on future dates, at Lovö vattenverk and Görvälnverket.

**Table S4**. List of 152 identified virus species that could be classified into indicated 76 virus families or orders and with known size and host and the group they were classified as

| **Size(nm)** | **Virus** | **Family or Class** | **Size(nm)** | **Host/group** |
| --- | --- | --- | --- | --- |
| 1-60 | Limestonevirus | *Ackermannviridae* | 60 | *Enterobacteriacea/*1 |
|  | Dickeya phage | *Ackermannviridae* | 60 | *Dickeya* *Pectobacterium/*2^d^ |
|  | Guanarito mammarenavirus | *Arenaviridae* | 50-200 | Mammals/3 |
|  | Pectobacterium phage | *Chaseviridae* | 53-60 | *Pectobacteriaceae/*2^d^ |
|  | Caulobacter phage | *Leviviridae* | *26* | Caulobacter/1 |
|  | Citrobacter phage | *Autographviridae* | 60 | Citrobacter/2^b^ |
|  | Enterobacter phage | *Tectiviridae* | 60-66 | *Enterobacteriacea/*1 |
|  | Klebsiella phage | *Autographviridae* | 53-60 | Klebsiella/1 |
|  | Morganella phage | *Autographviridae* | 60 | Morganella/1 |
|  | Salmonella phage | *Autographviridae* | 60 | Salmonella/1 |
|  | Aquamicrobium phage | *Autographviridae* | 60 | Aquamicrobium/1 |
|  | Przondovirus KP32 | *Autographviridae* | 60 | Klebsiella/1 |
|  | Providencia phage | *Straboviridae* | 60 | *Enterobacteriacea/*1 |
|  | Lymantria dispar multiple Nucleopolyhedrovirus | *Baculoviridae* | 30x300 | Arthropods/3 |
|  | Antheraea pernyi nucleopolyhedrovirus | *Baculoviridae* | 30x300 | Arthropods/3 |
|  | Mythimna unipuncta granulovirus | *Baculoviridae* | 30x300 | Invertebrates/3 |
|  | Rosebushvirus | *Bclasvrinae* | 66 | Mycobacterium/1 |
|  | Sweet potato C6 virus | *Betaflexviridae* | 13 | Plants/3 |
|  | Cucumber green mosaic virus | *Bromoviridae* | 30 | Plants/3 |
|  | Primate norovirus | *Caliciviridae* | 40 | Mammals/3 |
|  | Brucella phage | *Caudoviricetes* | 60 | Brucella/2^b^ |
|  | Cyanophage | *Caudoviricetes* | 55-90 | Cyanobacteria/1 |
|  | Delftia phage | *Caudoviricetes* | 60-85 | Delftia/1 |
|  | Rhodococcus phage | *Caudoviricetes* | 43-60 | Rhodococcus/1 |
|  | Shewanella phage | *Chaseviridae* | 53-65 | Shewanella/1 |
|  | Clostridium phage | *Guelinviridae* | 40-44 | Clostridium/1 |
|  | Flavobacterium phage | *Finnlakeviridae* | 59 | Flavobacterium/1 |
|  | Microcystis phage | *Myoviridae* | 74 | Microcystis/1 |
|  | Omegavirus | *Caudoviricetes* | 100 | Mycobacterium/1 |
|  | Pelagibacter phage | *Ubiqueviridae* | 40 | Bacterioplankton/1 |
|  | Ruegeria phage | *Caudoviricetes* | 47-55 | Rugeria/1 |
|  | Yersinia phage | *Chaseviridae* | 53-65 | Yersinia/2^b^ |
|  | Geobacillus phage | *Siphoviridae* | 53x30 | Geobacillus/1 |
|  | Mycobacterium phage | *Myoviridae* | 78-111 | Mycobacterium/1 |
|  | Arthrobacter phage | *Siphoviridae* | 53x30 | Arthrobacter/1 |
|  | White spot RNA virus | *Birnaviridae* | 55-60 | Invertebrates/3 |
|  | Sinorhizobium phage | *Myoviridae* | 73-84 | Sinorhizobium/2^d^ |
|  | Proteus phage | *Chaseviridae* | 53-65 | Proteus/1 |
|  | Prochlorococcus phage | *Kyanoviridae* | 60 | Prochlorococcus/1 |
|  | Cassava associated gemycircularvirus | *Genomoviridae* | 38 | Plants/3 |
|  | Ralstonia phage | *Inoviridae* | 10x600 | Ralstonia/1 |
|  | Xanthomonas phage | *Inoviridae* | 10x600 | Xanthomonas/2^d^ |
|  | Lymphocystis disease virus | *Iridoviridae* | 190-200 | Vertebrates/3 |
|  | Kolesnikvirus | *Myoviridae* | 45-65 | Erwinia/2^d^ |
|  | Tenacibaculum phage | *Kungbxnavirus* | 60 | Tenacibaculum/2^c^ |
|  | Sphingobium phage | *Lacusarxvirus* | 50x130 | Sphingobium/1 |
|  | Leicestervirus | *Leichestervirus* | 42x54 | Clostridium/1 |
|  | Sulfolobus islandicus filamentous virus | *Lipothrixviridae* | 25x300 | Sulfolobales/1 |
|  | Sulfolobus turreted icosahedral virus | *Turriviridae* | 60-75 | Sulfolobales/1 |
|  | Pseudomonas phage | *Mesyanzhinovviridae* | 50-72 | Pseudomonas/1 |
|  | Gokushovirinae | *Microviridae* | 25 | Gokushovirus/1 |
|  | Liberibacter phage | *Microviridae* | 25 | Liberibacter/2^d^ |
|  | Microbacterium phage | *Minunavirus* | 50 | Microbacteria/1 |
|  | Boolarra virus | *Nodaviridae* | 25-33 | Arthropods/3 |
|  | Deltapapillomavirus | *Papillomaviridae* | 55 | Mammals/3 |
|  | Human papillomavirus | *Papillomaviridae* | *55* | Mammals/3 |
|  | Porcine parvovirus | *Parvoviridae* | 23-28 | Mammals/3 |
|  | Porcine bocavirus | *Parvoviridae* | 23-28 | Mammals/3 |
|  | Propionibacterium phage | *Paulinoviridae* | 12x830 | Propionibacterium/2^b^ |
|  | Serratia phage | *Caudoviricetes* | 60 | Serratia/1 |
|  | Phietavirus | *Phiteavirus* | 50 | Staphylococcus/2^b^ |
|  | Streptomyces phage | *Picardvirus* | 53 | Streptomyces/1 |
|  | Duck aalivirus 1 | *Picornaviridae* | 30 | Birds/3 |
|  | Hubei picorna-like virus | *Marnaviridae* | *22-35* | Arthropods/3 |
|  | Synechococcus phage | *Kyanoviridae* | *47-65* | Synechococcus/1 |
|  | Helicobacter phage | *Podoviridae* | 60 | Helicobacter pylori/2^b^ |
|  | Sweet potato feathery mottle virus | *Potyviridae* | 15x700 | Plants/3 |
|  | Beihai narna-like virus | *Marnaviridae* | 22-35 | Invertebrates/3 |
|  | Sanxia picorna-like virus | *Marnaviridae* | 22-35 | Invertebrates/3 |
|  | Shahe picorna-like virus | *Marnaviridae* | 22-35 | Invertebrates/3 |
|  | Wenzhou picorna-like virus | *Marnaviridae* | 22-35 | Invertebrates/3 |
|  | Beihai tombus-like virus | *Riboviria* | 32-35 | Invertebrates/3 |
|  | Wenzhou channeled applesnail virus | *Marnaviridae* | 22-35 | Invertebrates/3 |
|  | Beihai picorna-like virus | *Marnaviridae* | 22-35 | Arthropods/3 |
|  | Beihai sobemo-like virus | *Riboviria* | 20-34 | Invertebrates/3 |
|  | Hubei leech virus | *Marnaviridae* | 22-35 | Invertebrates/3 |
|  | Hubei narna-like virus | *Riboviria* | 40 | Arthropods/3 |
|  | Hubei tombus-like virus | *Riboviria* | 40 | Arthropods/3 |
|  | Streptococcus phage | [*Aliceevansviridae*](https://www.ncbi.nlm.nih.gov/Taxonomy/Browser/wwwtax.cgi?mode=Undef&id=3044455&lvl=3&keep=1&srchmode=1&unlock) | 58-60 | Streptococcus/2^c^ |
|  | Bacillus phage | *Salasmviridae* | 45x54 | Bacillus/1 |
|  | Stx2-converting phage | *Sepvirinae* | 50 | Escherichia coli/2^a^ |
|  | Salicola phage | *Siphoviridae* | 80 | Salicola/1 |
|  | Lactococcus phage | *Ceudovirus* | 50 | Lactococcus/2^b^ |
|  | Acinetobacter phage | *Straboviridae* | 50 | Acinetobacter/1 |
|  | Aeromonas phage | *Chaseviridae* | 52-56 | Aeeromonas/1 |
|  | Escherichia phage | *Chaseviridae* | 52-56 | Escherichia coli/2^a^ |
|  | Stenotrophomonas phage | *Inoviridae* | 10x600 | Stenotropomonas/1 |
|  | Vibrio phage | *Inoviridae* | 10x600 | Vibrio/1 |
|  | Mosigvirus | *Straboviridae* | 86-120 | Escherichia coli/2^a^ |
|  | Listeria phage | *Trabyvirinae* | 50 | Listeria/1 |
|  | Alphabaculovirus | *Baculoviridae* | 30x300 | Arthropods/3 |
|  | Burkholderia phage | *Stanholtvirus* | 50 | Burkholderia/1 |
|  | Rhizobium phage | *Caudioviricetes* | 45-60 | Rhizobium/2^d^ |
|  | Campylobacter phage | *Caudioviricetes* | 45-60 | Campylobacter/2^c^ |
|  | Cronobacter phage | *Straboviridae* | 50 | Cronobacter/1 |
|  | Gordonia phage | *Zierdtviridae* | 60 | Gordonia/1 |
|  | Dinoroseobacter phage | *Schitoviridae* | 50 | Dinoroseobacter/1 |
|  | Pseudoalteromonas phage | *Zobellviridae* | 60-66 | Pseudoaltermonas/1 |
| 61-120 | Duck adenovirus | *Adenoviridae* | 90 | Vertebrates/3 |
|  | Fowl aviadenovirus | *Adenoviridae* | 90 | Vertebrates/3 |
|  | Kallithea virus | *Nudiviridae* | 80x400 | Arthropods/3 |
|  | Borna disease virus | *Bornaviridae* | 80 | Mammals/3 |
|  | Bat coronavirus | *Coronaviridae* | 120 | Mammals/3 |
|  | Erwinia phage | *Demereceviridae* | 90 | Erwinia/2^d^ |
|  | Staphylococcus phage | *Herelleviridae* | 80 | Staphylococcus/2^b^ |
|  | Maverick-related virus | *Lavidaviridae* | 75 | Cafeteria roenbergensis virus/1 |
|  | Lymphocystis disease virus | *Iridoviridae* | 200 | Vertebrates/3 |
|  | Corynebacterium phage | *Caudioviricetes* | 120 | Corynebacterium/1 |
|  | White spot syndrome virus | *Nimaviridae* | 70-120 | Invertebrates/3 |
|  | Influenza virus | *Orthomyxoviridae* | 80-120 | Mammals/3 |
|  | Shamonda orthobunyavirus | *Peribunyaviridae* | 80-120 | Arthropods/3 |
|  | Campoletis sonorensis ichnovirus | *Polydnaviriformidae* | 85x330 | Arthropods/3 |
|  | Propionibacterium phage | *Paulinoviridae* | 12x620 | Propionibacterium/2^b^ |
|  | Methanothermobacter phage | *Leisingerviridae* | 55 | Methanothermobacter/1 |
|  | Canine mastadenovirus | *Adenoviridae* | 90 | Mammals/3 |
|  | Eilatvirus | *Togaviridae* | 60-70 | Invertebrates/3 |
|  | Thermus phage | *Caudioviricetes* | 70 | Archea/1 |
| >121 | Cyprinid herpesvirus | *Alloherpesviridae* | *150-200* | Vertebrates/3 |
|  | Pacmanvirus | *Asfarviridae* | *170-190* | Amoeba/1 |
|  | Ateline alphaherpesvirus | *Orthoherpesviridae* | *150-200* | Mammals/3 |
|  | Alcelaphine gammaherpesvirus | *Orthoherpesviridae* | *150-200* | Mammals/3 |
|  | Anguillid herpesvirus | *Alloherpesviridae* | *150-200* | Vertebrates/3 |
|  | Tokyovirus | *Marseilleviridae* | *250* | Amoeba/1 |
|  | Mimivirus | *Mimiviridae* | *500* | Amoeba/1 |
|  | Bovine alphaherpesvirus | *Orthoherpesviridae* | *150-200* | Mammals/3 |
|  | Bubaline alphaherpesvirus | *Orthoherpesviridae* | *150-200* | Mammals/3 |
|  | Gallid alphaherpesvirus | *Orthoherpesviridae* | *150-200* | Vertebrates/3 |
|  | Suid alphaherpesvirus | *Orthoherpesviridae* | *150-200* | Mammals/3 |
|  | Human gammaherpesvirus | *Orthoherpesviridae* | *150-200* | Mammals/3 |
|  | Macacine betaherpesvirus | *Orthoherpesviridae* | *150-200* | Mammals/3 |
|  | Macropodid alphaherpesvirus | *Orthoherpesviridae* | *150-200* | Mammals/3 |
|  | Equid alphaherpesvirus | *Orthoherpesviridae* | *150-200* | Mammals/3 |
|  | Human alphaherpesvirus | *Orthoherpesviridae* | *150-200* | Mammals/3 |
|  | Human betaherpesvirus | *Orthoherpesviridae* | *150-200* | Mammals/3 |
|  | Columbid alphaherpesvirus | *Orthoherpesviridae* | *150-200* | Vertebrates/3 |
|  | Macacine alphaherpesvirus | *Orthoherpesviridae* | *150-200* | Mammals/3 |
|  | Murid betaherpesvirus | *Orthoherpesviridae* | *150-200* | Mammals/3 |
|  | Pandoravirus | *Pandoraviridae* | *1000* | Amoeba/2 |
|  | Avian avulavirus | *Paramyxoviridae* | *300-500* | Vertebrates/3 |
|  | Acanthocystis turfacea chlorella virus | *Phycodnaviridae* | *220* | Algae/1 |
|  | Only Syngen Nebraska virus | *Phycodnaviridae* | *220* | Algae/1 |
|  | Orpheovirus | *Phycodnaviridae* | *220* | Amoeba/1 |
|  | Yellowstone lake phycodnavirus | *Phycodnaviridae* | *220* | Algae/1 |
|  | Cedratvirus | *Cedratviridae* | *1100* | Amoeba/2 |
|  | Amsacta moorei entomopoxvirus | *Poxviridae* | *250-300* | Arthropods/3 |
|  | Canarypox virus | *Poxviridae* | *250-300* | Birds/3 |
|  | Choristoneura fumiferana entomopoxvirus | *Poxviridae* | *250-300* | Arthropods/3 |
|  | Swinepox virus | *Poxviridae* | *250-300* | Mammals/3 |
|  | BeAn | *Poxviridae* | *250-300* | Mammals/3 |
|  | Molluscum contagiosum virus | *Poxviridae* | *250-300* | Mammals/3 |
|  | Cowpox virus | *Poxviridae* | *250-300* | Mammals/3 |
|  | Leptopilina boulardi filamentous virus | *Lefavirales* | 30x300 | Arthropods/3 |

2^a^ = Phages fecal indicators

2^b^ = Phages mammalian indicators

2^c^ = Phages vertebrate indicators

2^d^ = Phages plant indicators

**Table S5**. Number of viral contigs /1,000 L for the different sizes (nm) of viruses for each DWT

| **DWTPs** | **Size(nm)** | **2021** | | | | | | | | | | **2022** | | | | |
| --- | --- | --- | --- | --- | --- | --- | --- | --- | --- | --- | --- | --- | --- | --- | --- | --- |
|  |  | **March/April** | | **May/June** | | **July/August** | | **September/October** | | **November/December** | | | **January/February** | | **March/April** | |
| Lovö vattenverk |  | Inlet | Outlet | Inlet | Outlet | Inlet | Outlet | Inlet | Outlet | Inlet | Outlet | | Inlet | Outlet | Inlet | Outlet |
|  | 1-60 | 1,44E+07 | 50 353 | 1,83E+07 | 62 171 |  |  | 1,90E+07 | 16 738 | 920 | 3 524 | | 880 | 1 528 | 1,00E+06 | 14 182 |
|  | 61-120 | 80 | - | 400 | 48 |  |  | 200 | 492 | - | - | | 40 | - | 4,56E+06 | 1 212 |
|  | >121 | 1,48E+06 | 14 706 | 4,12E+06 | 5 486 |  |  | 240 | 615 | 40 | 190 | | 40 | 102 | 80 | 12 848 |
| Görvälnverket | 1-60 | 8,04E+06 | 12 899 | 3,47E+07 | 644 | 5,80E+06 | 907 | 1,58E+07 | 40 624 |  |  | | 7,44E+06 | 1 282 | 720 | 637 |
|  | 61-120 | 80 | 628 | 640 | - | 80 | - | 4,52E+06 | 274 |  |  | | 360 | 49 | 40 | - |
|  | >121 | 4,20E+06 | 7 198 | 5,88E+06 | - | 240 | 2 268 | 4,12E+06 | 14 136 |  |  | | 920 | 247 | 80 | - |
| Borgs  vattenverk | 1-60 | 449 604 | 24 673 | 77 674 | 23 689 | 57 064 | 14 854 | 348 | 6 993 | 240 | 2 872 | | 21 116 | 12 808 |  |  |
|  | 61-120 | 8 443 | 602 | 5 441 | 347 | 19 945 | 398 | 400 | 1 589 | 77 241 | 160 | | 603 | 261 |  |  |
|  | >121 | 325 066 | 42 606 | 1 163 | 11 902 | 277 | 398 | 2 | 318 | 8 276 | 7 499 | | 603 | 261 |  |  |
| Kvarnagårdens  Vattenverk | 1-60 | 938 667 | 20 256 | 707 483 | 1 511 | 527 568 | 13 932 | 512 | 7 721 | 379 487 | 27 714 | | 763 399 | 1 466 |  |  |
|  | 61-120 | 560 | 2 906 | 103 401 | 923 | 47 568 | 2 336 | 42 667 | 345 | 5 641 | 1 853 | | 62 745 | 183 |  |  |
|  | >121 | 48 | 5 983 | 29 932 | - | 60 541 | 433 | 48 | 657 | - | 1 692 | | 580 392 | 183 |  |  |
| Vombverket | 1-60 | 1,51E+06 | 183 338 | 221 | 4515 | 5,52E+06 | 1 104 | 790 | 1 003 | 20 642 | 2 432 | | 503 965 | 21 211 |  |  |
|  | 61-120 | 3 451 | 8 902 | 0 | 72 | 18 349 | 216 | 80 | 201 | 4 587 | - | | 7 048 | 563 |  |  |
|  | >121 | 75 294 | 818 | 221 | 1792 | 1,50E+06 | 4 005 | 2,67E+06 | 5 617 | 25 229 | - | | 7 048 | 3 942 |  |  |
| Ringsjöverket | 1-60 | 67 297 | 6 127 |  | 18758 | 24 385 | 263 | 1223841 | 6446 | 1,14E+06 | 979 | | 609 649 | 2 914 |  |  |
|  | 61-120 | 10 727 | - |  | 296 | 562 | 64 | 105960 | 0 | 13 029 | - | | 4 386 | 139 |  |  |
|  | >121 | 8 243 | - |  | 3258 | 1 124 | 449 | 222517 | 817 | 291 857 | 490 | | 13 158 | 139 |  |  |

**Table S6.** Number of viral contigs/1,000 L for the viruses classified based on the host for each DWTP. **Group 1** are viruses with hosts that may be present during the purification process, **Group 2** are viruses (mainly bacteriophages) with hosts probably not present during the purification process and **Group 3** are viruses with no host present during the purification process

| **DWTPs** | **Size(nm)** | **2021** | | | | | | | | | | | **2022** | | | | |
| --- | --- | --- | --- | --- | --- | --- | --- | --- | --- | --- | --- | --- | --- | --- | --- | --- | --- |
|  |  | **March/April** | | **May/June** | | **July/August** | | **September/October** | | | **November/December** | | **January/February** | | **March/April** | | |
| Lovö  Vattenverk | Type of host | Inlet | Outlet | Inlet | Outlet | Inlet | Outlet | | Inlet | Outlet | Inlet | Outlet | Inlet | Outlet | Inlet | Outlet | |
|  | Group 1 | 1,39E+07 | 55 647 | 1,54E+07 | 53 371 |  |  | | 1,68E+07 | 14 523 | 840 | 3 238 | 520 | 306 | 880 | 24 364 | |
|  | Group 2 | 1,56E+06 | 5 294 | 1,28E+06 | 11 429 |  |  | | 2,56E+06 | 2 585 | 80 | 476 | 120 | 102 | 160 | 3 515 | |
|  | Group 3 | 440 | 4 118 | 6,16E+06 | 7 657 |  |  | | 120 | 738 | 40 | - | 320 | 1 222 | 4,60E+06 | 364 | |
| Görvälnverket | Group 1 | 1,13E+07 | 17 391 | 3,53E+07 | 403 | 5,44E+06 | 5,261 | | 8,88E+06 | 52 153 |  |  | 6,76E+06 | 1 035 | 240 | 530 | |
|  | Group 2 | 1,00E+06 | 2 222 | 4,68E+06 | 201 | 560 | 907 | | 3,16E+06 | 2 196 |  |  | 1,36E+06 | 197 | 160 | 80 | |
|  | Group 3 | 40 | 1 111 | 1,20E+06 | 40 | 120 | 517 | | 1,24E+07 | 686 |  |  | 600 | 345 | 440 | 27 | |
| Borgs  vattenverk | Group 1 | 711 346 | 59 816 | 82 049 | 32 009 | 48 199 | 11 538 | | 28 | 4 662 | 222 069 | 26 645 | 19 306 | 12 024 |  |  | |
|  | Group 2 | 71 768 | 6 379 | 1 067 | 1 733 | 9 972 | 4 111 | | 76 | 3 921 | 26 207 | 9 254 | 3 017 | 1 176 |  | |  |
|  | Group 3 | - | 1 685 | 2 027 | 2 196 | 21 607 | - | | 16 | 318 | 77 241 | 479 | - | 131 |  | |  |
| Kvarnagårdens  vattenverk | Group 1 | 928 | 24 188 | 718 367 | 1 931 | 514 595 | 14 624 | | 533 333 | 8 131 | 358 974 | 17 966 | 1 260 131 | 1 466 |  | |  |
|  | Group 2 | 608 | 453 | 87 075 | 504 | 69 189 | 1 644 | | 58 667 | 3 039 | 76 923 | 7 654 | 62 745 | 183 |  | |  |
|  | Group 3 | 10 667 | 427 | 304 762 | - | 51 892 | 433 | | 10 667 | 657 | - | 5 639 | 8 366 | 183 |  | |  |
| Vombverket | Group 1 | 1,36E+06 | 155 188 |  |  | 3,23E+06 | 6 711 | | 1,21E+06 | 12 839 | 34 404 | 2 258 | 264 317 | 10 136 |  | |  |
|  | Group 2 | 247 843 | 42 827 |  |  | 1,03E+06 | 6 602 | | 150 | 802 | 6 881 | 174 | 248 458 | 5 819 |  | |  |
|  | Group 3 | 18 824 | 2 406 |  |  | 2,79E+06 | 1 948 | | 2,18E+06 | 2 207 | 9 174 | - | 5 286 | 9 761 |  | |  |
| Ringsjöverket | Group 1 | 647 | 1 245 |  |  | 21 464 | 2 245 | |  |  | 917 264 | 490 | 45 614 | 2 636 |  | |  |
|  | Group 2 | 20 663 | 1 436 |  |  | 3 708 | 449 | |  |  | 18 241 | 490 | 201 754 | 416 |  | |  |
|  | Group 3 | 903 | - |  |  | 899 | 449 | |  |  | 349 186 | 490 | 8 772 | 139 |  | |  |

**Supplementary Figure 1.** Number of viral contigs identified using BLAST in CLC and geNomad across drinking water treatment plants, presented for inlet (raw water) and outlet (drinking water) samples.
